# Real-time monitoring of the sugar sensing in *Saccharomyces cerevisiae* indicates endogenous mechanisms for xylose signaling

**DOI:** 10.1186/s12934-016-0580-x

**Published:** 2016-10-24

**Authors:** Daniel P. Brink, Celina Borgström, Felipe G. Tueros, Marie F. Gorwa-Grauslund

**Affiliations:** Applied Microbiology, Department of Chemistry, Lund University, P.O. Box 124, 22100 Lund, Sweden

**Keywords:** *Saccharomyces cerevisiae*, Biosensor, Sugar sensing, Signaling, Xylose, GFP, cAMP/PKA, Snf3p/Rgt2p, SNF1/Mig1p, Flow cytometry

## Abstract

**Background:**

The sugar sensing and carbon catabolite repression in Baker’s yeast *Saccharomyces cerevisiae* is governed by three major signaling pathways that connect carbon source recognition with transcriptional regulation. Here we present a screening method based on a non-invasive in vivo reporter system for real-time, single-cell screening of the sugar signaling state in *S. cerevisiae* in response to changing carbon conditions, with a main focus on the response to glucose and xylose.

**Results:**

The artificial reporter system was constructed by coupling a green fluorescent protein gene (*yEGFP3*) downstream of endogenous yeast promoters from the Snf3p/Rgt2p, SNF1/Mig1p and cAMP/PKA signaling pathways: *HXT1p/2p/4p; SUC2p, CAT8p; TPS1p/2p* and *TEF4p* respectively. A panel of eight biosensors strains was generated by single copy chromosomal integration of the different constructs in a W303-derived strain. The signaling biosensors were validated for their functionality with flow cytometry by comparing the fluorescence intensity (FI) response in the presence of high or nearly depleted glucose to the known induction/repression conditions of the eight different promoters. The FI signal correlated with the known patterns of the selected promoters while maintaining a non-invasive property on the cellular phenotype, as was demonstrated in terms of growth, metabolites and enzyme activity.

**Conclusions:**

Once verified, the sensors were used to evaluate the signaling response to varying conditions of extracellular glucose, glycerol and xylose by screening in 96-well microtiter plates. We show that these yeast strains, which do not harbor any recombinant pathways for xylose utilization, are lacking a signaling response for *extracellular* xylose. However, for the *HXT2p/4p* sensors, a shift in the flow cytometry population dynamics indicated that *internalized* xylose does affect the signaling. These results suggest that the previously observed effects of this pentose on the *S. cerevisiae* physiology and gene regulation can be attributed to xylose and not only to a lack of glucose.

**Electronic supplementary material:**

The online version of this article (doi:10.1186/s12934-016-0580-x) contains supplementary material, which is available to authorized users.

## Background

Baker’s yeast *Saccharomyces cerevisiae* can grow naturally in a variety of niches, ranging from plants and ripening fruit to soil and insect guts, that are diverse in nutrient type and content [1, 2]. The cellular uptake and metabolism of carbon sources in this yeast are regulated by a complex network of sensing and signaling cascades which allow the cells to recognize and respond to variations in the environmental carbon availability and to reprogram the phosphorylation and metabolite patterns and transcription levels accordingly [3–6]. Despite its broad variability in sensing and utilizing different carbon sources, wild type *S. cerevisiae* cannot efficiently utilize pentoses such as xylose and arabinose, and although endogenous genes for xylose utilization are present in the genome, they are inadequately expressed to support growth [7]. In addition, this yeast exhibits strong carbon catabolite repression on metabolism of alternative carbon sources when cultivated on glucose, its favored carbon source [6]. *S. cerevisiae* has become a eukaryotic model organism for studies in this field, and the signaling responses to glucose and other alternative fermentable carbon sources such as sucrose, maltose and galactose in this yeast are well-known [3, 6]. The signaling response to xylose, however, is not.

By virtue of its robustness, manageability and high genetic manipulability, *S. cerevisiae* has become an imperative protagonist in industrial bioprocesses and is a promising host for production of value-added chemicals from lignocellulosic biomass [8, 9]. However, a major research challenge in establishing lignocellulosic biomass as a sustainable feedstock for this yeast is that the xylose stream cannot yet be fully valorized—which is a particular issue as xylose is the second most abundant sugar in lignocellulosic hydrolysates [8, 9]. Although *S. cerevisiae* has been successfully engineered for pentose utilization by introduction of exogenous pathways from other yeasts [10–12], growth rates and productivity are significantly lower on this sugar compared with glucose and thus not industrially competitive [9]. In fact, the recombinant strains, despite being successfully engineered to utilize xylose, do not seem to recognize this carbon source as a fermentable sugar, as has been implied in multiple studies [13–19]. Taken together, these advances suggest something is lacking in the sensing and signaling of xylose in *S. cerevisiae*, and that this is a plausible bottleneck that has to be overcome in order to improve productivity of e.g. ethanol from xylose.

**Fig. 1:**
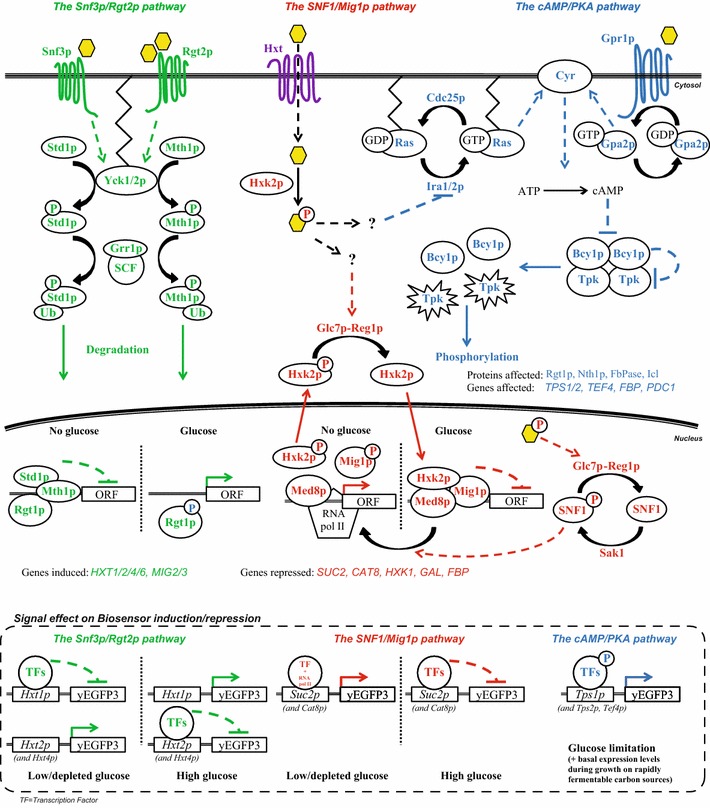
The three major pathways governing sugar perception and signaling in *S. cerevisiae*. The Snf3p/Rgt2p pathway (*green*) sensing extracellular glucose (*yellow* hexagons), the SNF1/Mig1p pathway (*red*) responding to phosphorylated intracellular glucose and the cAMP/PKA pathway (*blue*) sensing both extracellular and metabolized hexoses. *Circles* with *P* indicate phosphorylated targets and the *color of the letter* represent the pathway performing the phosphorylation; *Ub* indicates ubiquitination; *question marks* indicate currently unknown mechanisms; *star shape* indicates activation of Tpk. *Solid arrows* describe reaction steps/transport; *dashed arrows* with *arrowheads* indicate activation and *dashed arrows* with *bars* indicate repression/inhibition

Sugar sensing in *S. cerevisiae* is governed by three cross-talking signaling pathways (Fig. 1): the Snf3p/Rgt2p pathway senses extracellular hexoses and induces transcription of an array of hexose transporters (*HXT1*-*17*) [3, 20, 21]; the SNF1/Mig1p pathway (here represented by *SUC2* and *CAT8*) is a conveyor of catabolite repression by internalized glucose [6], and regulates induction of alternative carbon sources (including e.g. ethanol, glycerol and galactose) during glucose depletion by a not entirely elucidated interaction with Hexokinase isoenzyme 2 (Hxk2p; see Fig. 1) [22–24]; finally, the cAMP/PKA pathway responds to both internalized and external glucose through the Ras1p/2p paralogs or the Gpr1p/Gpa2p complex, respectively, and in accordance to this regulates the environmental stress response, cell cycle progression and homeostasis (here assessed by the *TPS1/2* genes) [5, 25]. Together, these pathways have evolved to support growth on versatile niches [2, 3]; consequently, *S. cerevisiae* gene expression is highly regulated by carbon-source dependent promoters (for a review see [26]).

In this study, we utilized a Green Fluorescent Protein (GFP) to design a panel of biosensors that allow for real-time single-cell evaluation of the sugar signaling state in *S. cerevisiae* using flow cytometry, which in turn comes with the possibility of detecting and analyzing population heterogeneities. Here, the promoter regions from eight genes (*HXT1p/2p/4p; SUC2p, CAT8p; TPS1p/2p*, *TEF4p*) under control by the Snf3/Rgt2, Snf1/Glc7 and RAS-cAMP-PKA pathways respectively were coupled to the established yeast reporter gene *yEGFP3* (yeast enhanced green fluorescent protein) [27] and were introduced in *S. cerevisiae*. Due to the non-invasive and heritable features of the small and inert GFP molecule [28], fluorescent protein biosensors have become a highly applied tool within the field of yeast biology [29–35]. The glucose repressed *JEN1* promoter (under control of the SNF1 complex) has previously been used as a biosensor to detect different concentrations of glucose based on GFP-intensity [36]; however, this study did not focus on any *S. cerevisiae* sensing of other sugars than glucose.

Despite recent attempts at resolving the Gordian knot of xylose sensing in *S. cerevisiae* by heterologous expression of bacterial xylose-responsive transcription regulators (XylRs) [37, 38], as well as engineering of carbon catabolite repression, by e.g. deletion of *MIG1/2,* in recombinant xylose utilizing strains [39], little is still known about how or even if xylose is sensed by this yeast. To our knowledge, the current study is the first time a fluorescent biosensor has been implemented to monitor the hexose-pentose signaling state of this yeast. Determining the position(s) in the cascade where the signal differentiates for xylose and glucose will allow us to find novel engineering targets for improving xylose uptake and utilization in *S. cerevisiae*.

## Methods

### Strains

The *S. cerevisiae* strains that were used in this study are listed in Table 1. The W303-1A strain [ATCC^®^ 208352], from which the engineered strains were derived, was purchased from ATCC (Manassas, VA, US). Competent *Escherichia coli* NEB5α (New England BioLabs, Ipswich, MA, US) was used for subcloning. *E. coli* DH5α containing the M3499 plasmid (Addgene plasmid #51674) was purchased from Addgene (Cambridge, MA, US). All strains were stored in 25% (v/v) glycerol at −80 °C. The yeast strains were maintained on Yeast Nitrogen Base (YNB)-glucose agar plates (6.7 g/L YNB w.o. amino acids, 20 g/L glucose, 20 g/L agar–agar) supplemented with amino acids (tryptophan 75 mg/L, histidine 125 mg/L, leucine 500 mg/L and uracil 150 mg/L [40] and adenine 100 mg/L [41]) depending on the strain requirements. Solid media cultivations were considered fresh for two weeks before new plates were streaked from the −80 °C stock.

**Table 1:** Yeast strains and plasmids used in the present study

Strain	Genotype	Reference
W303-1A	*MATa trp1*-*1 leu2*-*3,112 his3*-*11 ade2*-*1 ura3*-*1 can1*-*100*	[54]; ATCC^®^ 208352
TMB3700	W303-1A TRP1 HIS3, *ura3::M3499 (ADE2)*	This study
TMB3711	TMB3700; *can1::YIp211; SPB1/PBN1::YIp128*	This study
TMB3712	TMB3700, *can1::YIpGFP*-*Hxt1p; SPB1/PBN1::YIp128*	This study
TMB3713	TMB3700, *can1::YIpGFP*-*Hxt2p; SPB1/PBN1::YIp128*	This study
TMB3714	TMB3700, *can1::YIpGFP*-*Hxt4p; SPB1/PBN1::YIp128*	This study
TMB3715	TMB3700, *can1::YIpGFP*-*Suc2p; SPB1/PBN1::YIp128*	This study
TMB3716	TMB3700, *can1::YIpGFP*-*Cat8p; SPB1/PBN1::YIp128*	This study
TMB3717	TMB3700, *can1::YIpGFP*-*Tps1p; SPB1/PBN1::YIp128*	This study
TMB3718	TMB3700, *can1::YIpGFP*-*Tps2p; SPB1/PBN1::YIp128*	This study
TMB3719	TMB3700, *can1::YIpGFP*-*Tef4p; SPB1/PBN1::YIp128*	This study
CEN.PK 113-7D	*MATα, MAL2*-*8* ^*C*^ *, SUC2*	[76]; EUROSCARF collection

Plasmid	Relevant genotype	Reference
YIplac128	*AmpR; LEU2*	[50]
YIplac211	*AmpR; URA3*	[50]
YEpJK01	*YEplacHXT; URA3; GPD2p*-*yEGFP3*-*PGK1t*	[33]
YIpGFP	*YIplac211: yEGFP3*-*PGK1t*	This study
YIpGFP-HXT1p	*YIpGFP:HXT1p*-*yEGFP3*-*PGK1t*	This study
YIpGFP-HXT2p	*YIpGFP:HXT2p*-*yEGFP3*-*PGK1t*	This study
YIpGFP-HXT4p	*YIpGFP:HXT4p*-*yEGFP3*-*PGK1t*	This study
YIpGFP-SUC2p	*YIpGFP:SUC2p*-*yEGFP3*-*PGK1t*	This study
YIpGFP-CAT8p	*YIpGFP:CAT8p*-*yEGFP3*-*PGK1t*	This study
YIpGFP-TPS1p	*YIpGFP:TPS1p*-*yEGFP3*-*PGK1t*	This study
YIpGFP-TPS2p	*YIpGFP:TPS2p*-*yEGFP3*-*PGK1t*	This study
YIpGFP-TEF4p	*YIpGFP:TEF4p*-*yEGFP3*-*PGK1t*	This study
M3499	*AmpR; ura3::ADE2*	[52]

### Molecular biology methods

Standard molecular biology methods were used for all cloning procedures [42]. Restriction enzymes, Phusion DNA polymerase and T4 ligase were obtained from Thermo Scientific (Waltham, MA, US), with the exception of the *ZraI* restriction enzyme that was purchased from New England Biolabs (Ipswich, MA, US). PCR primers were ordered from Eurofins MWG Operon (Ebersberg, Germany); all primers that were used in this study can be found in Additional file 1: Table S1. Plasmid purification was done using the GeneJet plasmid MiniPrep kit (Thermo Scientific, Waltham, MA, US) and PCR products were purified using the GeneJET PCR purification kit from the same manufacturer. DNA extractions from agarose gels were made using the QIAquick gel extraction kit (Qiagen, Hilden, Germany). All genetic constructs were verified by sequencing (Eurofins MWG Operon, Ebersberg, Germany). The genomic DNA sequences from the CEN.PK and W303 strains that were used for the design of the cloning were retrieved from the *Saccharomyces* Genome Database (SGD; http://www.yeastgenome.org) [43]. Extraction of genomic yeast DNA was performed using the LiOAC-SDS method [44].

Competent *E. coli* NEB5α cells were prepared and transformed according to the method of Inoue and colleagues [45]; *E. coli* transformants were selected on Luria–Bertani medium (10 g/L tryptone, 5 g/L yeast extract and 10 g/L NaCl, agar 15 g/L) supplemented with 100 µg/mL ampicillin. *S. cerevisiae* cells were transformed according to the lithium acetate method [46] with addition of DMSO (10% v/v) prior to heat shock [47], and were streaked on selective medium.

### Construction of expression cassettes, targeting fragments and plasmids

The *yEGFP3*-*PGK1t* expression cassette containing the yeast enhanced GFP (*yEGFP3*) [27] and the *PGK1* terminator was PCR amplified from the YEpJK01 plasmid [33] using the *yEGFP*-*F1*-*KpnI* and *yEGFP*-*R1*-*SacI* primers. The promoter regions (ca 1 kb upstream of each ORF) from eight genes involved in the *S. cerevisiae* sugar signaling (*HXT1, HXT2, HXT4, SUC2, CAT8, TPS1, TPS2* and *TEF4*) were PCR amplified from genomic CEN.PK 113-7D DNA. The CEN.PK strain was originally intended to be used throughout the project, but following the construction of the biosensors plasmids it was found that this strain family has deficiencies in the cAMP/PKA pathway [48, 49], and the W303 strain was instead chosen for the sensor evaluation. Flanking *Sal*I and *Bam*HI restriction sites were introduced in each expression cassette respectively. Expression cassettes for the recovery of the tryptophan and histidine auxotrophies in W303-1A were also PCR amplified from CEN.PK 113-7D genomic DNA.

The YIpGFP plasmid was constructed by ligating the *yEGFP3*-*PGK1t* cassette into the integrative yeast shuttle vector YIplac211 [50] using the flanking *Kpn*I and *Sac*I restriction enzyme sites that were introduced when the cassette was PCR amplified from YEpJK01. The eight promoter-GFP reporter plasmids (YIpGFP-HXT1p, YIpGFP-HXT2p, YIpGFP-HXT4p, YIpGFP-SUC2p, YIpGFP-CAT8p, YIpGFP-TEF4p, YIpGFP-TPS1p and YIpGFP-TPS2p) were generated by ligation of each promoter cassette into YIpGFP through the *Sal*I and *Bam*HI sites respectively and the plasmids were transformed into *E. coli*. A schematic illustration of the plasmid map is found in Additional file 1: Figure S2, and all plasmids that were used in this study are listed in Table 1.

Targeting fragments for nested double homologous integration of the YIpGFP plasmids into the yeast genome were constructed by PCR amplification (previously described in e.g. [51]). A schematic illustration of the integration strategy is found in Additional file 1: Figure S1. The two targeting fragments for integration of the biosensor plasmids were generated by amplifying 300 bp regions in the 5′- and 3′-ends of the *CAN1* ORF from W303-1A genomic DNA with addition of 50 bp tails with homology to the flanks of the linear sequence of YIplac211 (after digestion with the blunt cutting *ZraI)*. The tail regions of the fragments were achieved by using 74 bp “tail” primers with 24 bp primers annealing to the chromosomal DNA. Likewise, 475 bp targeting fragments for integration of YIplac128 in the SPB1/PBN intergenic locus were generated using primers modified from Flagfeldt et al. [51].

### Yeast reporter strain construction

All yeast strains used in this study (Table 1) were constructed from the parental strain W303-1A. The strains were made prototrophic by sequential transformation with the *TRP1* and *HIS3* expression cassettes and the M3499 plasmid (*ura3::ADE2*) [52], followed by one of the eight GFP-reporter plasmids containing the *URA3* marker (YIpGFP-HXT1p, YIpGFP-HXT2p, YIpGFP-HXT4p, YIpGFP-SUC2p, YIpGFP-CAT8p, YIpGFP-TEF4p, YIpGFP-TPS1p, YIpGFP-TPS2p) respectively. Finally, the leucine auxotrophy was cured in all GFP-strains by integration of an empty YIplac128 (*LEU2* ^+^) plasmid in the *SPB1/PBN1* intergenic locus [51].

Since the uracil auxotrophy in W303-1A is caused by a single point mutation in the *Ura3*-*1* allele and is known to revert [53, 54], the *URA3* locus in the reporter strains was deleted to avoid the possibility of reversion to the wild type locus during the sequential transformations. This was made with the M3499 *ura3::ADE2* Disruptor Converter plasmid (Addgene plasmid #51674; a gift from David Stillman); digestion and red-white screening was performed according to the authors’ instructions [52].

The eight GFP-reporter plasmids and the empty YIplac128 plasmid (*LEU*2^+^) were linearized with *Zra*I and were integrated in the yeast genome by double homologous recombination with the targeting fragments described above. The correct integration of all plasmids was verified with PCR amplification of genomic DNA from transformant colonies.

### Cultivation conditions

Single colonies from the reporter strains were taken from YNB-glucose-plates and were cultivated in two steps prior to the experiments. The first step (pre–pre-cultivation) was performed in 50 mL conical tubes with 5 mL YNB-glucose20 medium (6.7 g/L YNB w.o. amino acids, 20 g/L glucose supplemented with potassium hydrogen phthalate buffer 50 mM pH 5.5) in order to gain sufficient biomass (10 h cultivation time). The second step consisted of a pre-cultivation with repressing conditions; depending on the induction/repression conditions of each GFP-reporter strain (Table 2) the liquid YNB-medium was complemented with either excess glucose (40 g/L; cultivated for 12 h in order not to deplete the glucose and induce the promoters) or ethanol (3% v/v; cultivated for 32 h to reach a sufficient biomass) in order to repress GFP-expression. For the *TEF4* biosensor (TMB3719) pre-culture, glucose 20 g/L and 24 h cultivation was used to achieve better repression. For the batch culture experiments, the pre-cultures were grown in 250 mL baffled shake flasks (25 mL YNB-glucose40 or YNB-EtOH3 medium) with an initial optical density (OD_620nm_) of 0.05. The pre-cultures for the microtiter plate experiments were performed in a similar manner, with the difference that 50 mL conical tubes (5 mL YNB-glucose40 or YNB-EtOH3 medium; initial OD_620nm_ = 0.05) were used. All yeast incubations were performed at 30 °C and 180 rpm unless otherwise specified.

**Table 2:** Summary of the documented induction/repression conditions for the *S. cerevisiae* promoters chosen for the GFP-reporter constructs

Promoter	Name/function	Signaling pathway	Induced/derepressed by	Repressed by	References
HXT1	Low-affinity hexose transporter	Snf3p/Rgt2p	High glucose (4% w/v)^a^	Low glucose (0.1% w/v)	[20]
HXT2	High-affinity hexose transporter	Snf3p/Rgt2p	Low glucose (0.1% w/v)	High glucose (4% w/v)	[20]
HXT4	High-affinity hexose transporter	Snf3p/Rgt2p	Low glucose (0.1% w/v)	High glucose (4% w/v); more glucose-repressed than HXT2	[20, 77]
SUC2	Invertase	SNF1/Mig1p	Low glucose (0.1% w/v)	High glucose (2% w/v) and depleted glucose (0% w/v)	[78]
CAT8	*Alternative carbon source response*-activator	SNF1/Mig1p	Low glucose (0.2% w/v)	High glucose (4% w/v)	[79]
TPS1	Trehalose-6-phosphate synthase (56 kD subunit)	cAMP/PKA	Glucose limitation, stress conditions (e.g. heat, nutrient starvation, oxidative stress)	High glucose; however, a basal expression level has been observed when growing on rapidly fermentable sugars	[80, 81]
TPS2	Trehalose-6-phosphate synthase (102.8 kD subunit)	cAMP/PKA			
TEF4	Translation elongation factor	cAMP/PKA	–	Stress conditions	[82, 83]

^a^Glucose 4% (w/v) corresponds to 40 g/l

The pre-cultures were harvested by centrifugation at 3000 rpm for 5 min (5810R centrifuge, Eppendorf, Hamburg, Germany) and the cell pellets were washed twice in 5 mL YNB-KHPthalate without glucose, were resuspended in 1 mL YNB-KHPthalate medium without glucose and were used to inoculate 1000 mL baffled shake flasks with a total end-volume of 100 mL and an initial OD_620nm_ of 0.5. From here on, aerobic batch cultivations were carried out in liquid YNB-KHPhtalate medium complemented with either high (20 g/L) or low (1 g/L) glucose concentrations (according to previous studies; Table 2). Aerobic conditions were chosen not to impact the GFP signal as the protein is known to require oxygen to mature [27]; furthermore, since this yeast is Crabtree positive [55], respiro-fermentative growth will occur during these conditions. Two biological replicates were performed for each strain and condition.

Microtiter plate based experiments were carried out in a similar fashion to the shake flask experiments. The pre-cultures were harvested in a benchtop centrifuge in 2 mL conical tubes at 2300 RCF for 2 min (5415R centrifuge, Eppendorf, Hamburg, Germany) and washed twice in 1.5 mL YNB-KHPthalate medium without glucose. The cells were inoculated in pre-sterilized 96 U-well microtiter plates (Microtest Plate 96 well R, Sarstedt, Nümbrecht, Germany) with a 250µL total volume per well and initial OD_620nm_ = 0.5. The microtiter plates were incubated at room temperature (24–25 °C) in microtiter plate shaker (IKA MS3 Basic, Staufen, Germany) at 800 rpm both during and in-between flow cytometry analysis since the flow cytometer could not be incubated. The single-cell fluorescence was evaluated in eight different conditions in YNB-KHPthalate medium with the following supplements: no glucose; glucose 1, 20 and 5 g/L; glycerol 3% (v/v); xylose 50 g/L; xylose 50 g/L with glucose 5 g/L; xylose 50 g/L with glycerol 3% (v/v). The autosampler was paused after half of the wells had been injected, and the remaining wells were mixed thoroughly by pipetting the liquid in each well up and down in order to counteract cell sedimentation. The strains were analysed in two biological replicates with two technical replicates per strain, condition and plate.

### Analyses and sampling procedures

#### Growth and metabolite profiles

Growth of the cell cultures were monitored by optical density (OD) at 620 nm using an Ultrospec 2100 Pro spectrophotometer (Amersham Biosciences, Uppsala, Sweden). Extracellular glucose, glycerol, acetate and ethanol were quantified with a Waters HPLC system (Mildford, MA, USA). The separation was performed with an HPX-87H ion exchange column (Bio-Rad, Hercules, CA, USA). The mobile phase consisted of 5 mM H_2_SO_4_ at a flow rate of 0.6 mL/min and a column temperature of 60 °C. A refractive index detector (RID-6a, Shimadzu, Kyoto, Japan) was used for detection. Growth and metabolite analyses were performed in technical duplicates for every sample.

#### Flow cytometry

The yeast GFP-reporter strains were evaluated for fluorescence intensity (FI) with a BD Accuri C6 flow cytometer equipped with a BD CSampler autosampler (Becton–Dickinson, NJ, US). A laser with an excitation wavelength of 488 nm was used and fluorescence emission levels were measured with a 533/30 bandpass filter. Quality control was performed prior to each experiment with Spherotech 8**-**peak and 6-peak validation beads (Becton–Dickinson, NJ, US). A flow rate of 14 μL/min and a core size of 10 μm were used when analysing cells. The threshold was set to 8000 at the forward scatter-height (FSC-H) channel and 100,000 events were collected per sample. Every cell sample was followed by an auto-sampler wash cycle and a 2 min injection of deionised water in order to minimize sample carryover between injections. For the microtiter plate cultivations, 10,000 events were collected per sample and the mean of the technical replicates was used in the data analysis of each biological replicate; this could be done since the technical replicates were highly consistent within each pair (in terms of standard deviation). The raw data has been deposited at FlowRepository (https://flowrepository.org/) [56] with accession numbers FR-FCM-ZZRA, FR-FCM-ZZRB, FR-FCM-ZZRC, FR-FCM-ZZRD and FR-FCM-ZZRE.

Flow cytometry raw data was exported from the Accuri software as fcs-files and was processed and analyzed with the Knijnenburg morphology correction model [57] in Matlab (Release R2015a, The MathWorks, Inc., Natick, MA, US) using the FCS data reader function (version 28 May 2014; L. Balkay, University of Debrecen, Hungary; downloaded from http://www.mathworks.com/matlabcentral). FlowJo (v10; Treestar, Inc., San Carlos, CA) was used to produce overlay histograms for certain visualisation purposes. A custom, in-house Matlab script was developed to allow high-throughput, automated signal-to-cell size normalization and visualization of the population average based on the geometrical mean of the FL1-H channel (GFP) histograms. The script identifies .fcs raw data for each time point and calls the Knijnenburg model to normalize the fluorescence intensity (FI) to cell size and outputs plots of the normalized FI over time for each strain. Non-Gaussian histograms were predominantly observed in the raw FI data from the glucose 1 g/L cultures; it was however found that this was caused by size heterogeneities in the cultures, as the morphology-normalized data proved to compensate for skewed distributions in the in-data. A complementary custom in-house Python script was developed to facilitate renaming of file names and sorting in the required folder hierarchy in order for the custom Matlab script to function properly. See also Additional file 1 for a more detailed description of the custom scripts. The custom scripts and operation instructions have been deposited on Github (https://github.com/tmbyeast/Flow-cytometry-tools), and this is also where possible future updates will be stored. Users will have to download the Knijnenburg model separately according to the authors’ instructions [57].

A mean, time-independent FI baseline [corresponding to the autofluorescence of the negative control TMB3711 at the FL1-H channel and excitation wavelength (488 nm)] was calculated as the average FI of the biological replicates for the glucose 20 and 1 g/L cultures (four replicates in total). The baseline was only used to indicate the approximate autofluorescence of the utilized strains in order to facilitate comparisons with the FI induction/repression patterns of the biosensor strains (TMB3712-3719).

Matlab (Release R2015a, The MathWorks, Inc., Natick, MA, US) was also used to perform one-way ANOVA tests (*anova1*) coupled with a multiple comparison test (*multcompare*).

#### RT-qPCR assay

Culture samples for mRNA analysis were quenched in cold methanol (−80 °C; 1.4 mL methanol per 1 mL cell culture [58]) and centrifuged at 1800 RCF and 0 °C for 5 min. The supernatants were decanted and the cell pellets were stored in −80 °C. RNA was extracted using the RNeasy Mini Kit (Qiagen, Hilden, Germany) using mechanical lysis: the quenched cell samples were resuspended in RLT buffer previously mixed with 2-mercaptoethanol, and were lysed by bead beating in a Precellys 24 (6500 rpm, 3 cycles á 60 with 30 s pauses in-between cycles; Bertin Technologies, France) with a Cryolys temperature controller (Bertin Technologies, France) cooled with liquid nitrogen. Residual DNA was removed from the extract with rDNase I (Ambion, Life Technologies, Carlsbad, CA, US). Conventional PCR was used to verify that no residual DNA was left in the RNA extract. The RNA content was quantified using a BioDrop (BioDrop Ltd, Cambridge, UK). The superscript IV Reverse Transcriptase kit and Oligo(DT)_20_ primers (Invitrogen, Life Technologies, Carlsbad, CA, US) were used to synthesise cDNA from extracted RNA (using 0.5 mg/mL of RNA extract per sample). RT-qPCR was performed using the Ex Taq DNA polymerase kit (Takara Bio, Kusatsu, Shiga, Japan), EvaGreen dye (Biotium, Hayward, CA, US), bovine serum albumin (20 mg/mL; Thermo Scientific, Waltham, MA, US) and a LightCycler 2.0 (Roche Life Science, Basel, Switzerland). Quantification cycle (C_q_)-values and melting curve analyses were determined using the LightCycler software 4.1 (Roche Life Science, Basel, Switzerland). *ACT1*, *UBC6* and *TAF10* were evaluated as reference gene candidates [59]. Due to its stability during the evaluated conditions, *ACT1* was chosen as the reference gene (using primers *ACT1_F*/*ACT1_R*); *yEGFP3* and *SUC2* were used as target genes (primers *yEGFP3_F1790_RT*/*yEGFP3_R1918_RT* and *SUC2_F263_RT*/*SUC2_R397_RT* respectively). The following RT-qPCR program was used to analyze all three genes: denaturation 95 °C 2 min; 45 cycles of 95 °C 10 s, 55 °C 10 s, 72 °C 30 s; melting curve analysis: 50 °C 1 min hold time, ramp to 95 °C with 0.05 °C/s; cooling: 40 °C, 30 s. Standard curves for calculation of RT-qPCR efficiency and assessment of gene relative expressions were performed according to the Pfaffl method [60]. Each sample was analyzed in biological and technical triplicates.

#### Invertase enzyme assay

Enzymatic activity of invertase (EC: 3.2.1.26; encoded by *SUC2*) was assessed in yeast cell extracts according to previous protocols [61, 62] with the exception that the carcinogenic *o*-dianisidine was substituted with 4-aminophenazone/phenol [63]. A detailed description of the adapted protocol and calculation of the specific invertase activity can be found in Additional file 1. Assays were performed in biological triplicates.

#### Sampling procedures

The 100 mL batch shake flask cultures were sampled for OD (500µL) and FI (200µL) measurements every hour, for metabolite concentrations (2 mL) every second h for the first 8 h and at 24 h, and for mRNA (4 mL) at 0, 15, 30, 45 and 60 min. Enzyme assay samples were collected every 30 min for the first 3 h from cultures grown in 1 g/L glucose. The sample volumes were designed to minimize the influence of sampling on the culture, by having at least 50% of the starting volume left in the flask after the final sample point. The microtiter plates were sampled by the Accuri autosampler at 0, 3 and 6 h. Each run lasted ~90 min (a technical limitation of the flow cytometer).

## Results

### Design and construction of the yeast biosensor strains

A panel of eight *S. cerevisiae* sugar-responsive biosensor strains were constructed by coupling select promoters from the three main sugar sensing pathways in this yeast (Snf3p/Rgt2p, SNF1/Mig1p and cAMP/PKA; Fig. 1) with a green fluorescent protein (GFP) gene followed by genomic integration (one biosensor/strain). The biosensor expression cassettes were produced by cloning of endogenous *S. cerevisiae* promoters circa 1 kb upstream from the *HXT1/2/4, SUC2, CAT8, TPS1/2* and *TEF4* genes in front of the *yEGFP3* cassette [27] in the YIplac211 vector [50] (cf. Additional file 1: Figure S2). The promoter length was roughly based on the known binding motifs of these regions (Additional file 1: Table S2). The promoters were chosen for their known interactions with said pathways (Table 2) [3, 6], and/or based upon changes in their transcriptomics profiles in deletion mutant strains, compared to the wild type [64].

In vivo recombination and double homologous integration of nested DNA fragments [51] was used to integrate the biosensor cassettes in single-copy in the TMB3700 strain, that in turn was derived from *S. cerevisiae* W303-1A (Table 1). In addition to the eight biosensor strains, a ninth strain was integrated with an empty YIp211 plasmid (i.e. lacking *yEGFP3* and promoter) and was used as a negative control in order to measure the cellular auto-fluorescence at the GFP emission wavelength.

### Validation of the induction/repression patterns of the sugar signaling biosensors

In order to allow for assessment of the *S. cerevisiae* signaling response to xylose, the response of the eight biosensors were first validated on high (20 g/L) and low (1 g/L) glucose by comparing the fluorescence intensity (FI) with the already well documented induction/repression patterns of the eight promoters (Table 2). Flow cytometry was used to measure the FI at a single-cell level; this methodology was chosen for the biosensor protocols since it allows for identification of possible population heterogeneities (subpopulations) and can later on be used with cell sorting experiments (i.e. by FACS: fluorescence-activated cell sorting); the non-pooled, single-cell approach of this methodology will therefore be able to generate data that complements traditional pooled-sample strategies like transcriptomics and RT-qPCR. KHPthalate buffer was added to the culture media to minimize pH stress on the cell, as stress conditions are known to induce/repress *TPS1p/2p* and *TEF4p* (Table 2).

**Fig. 2:**
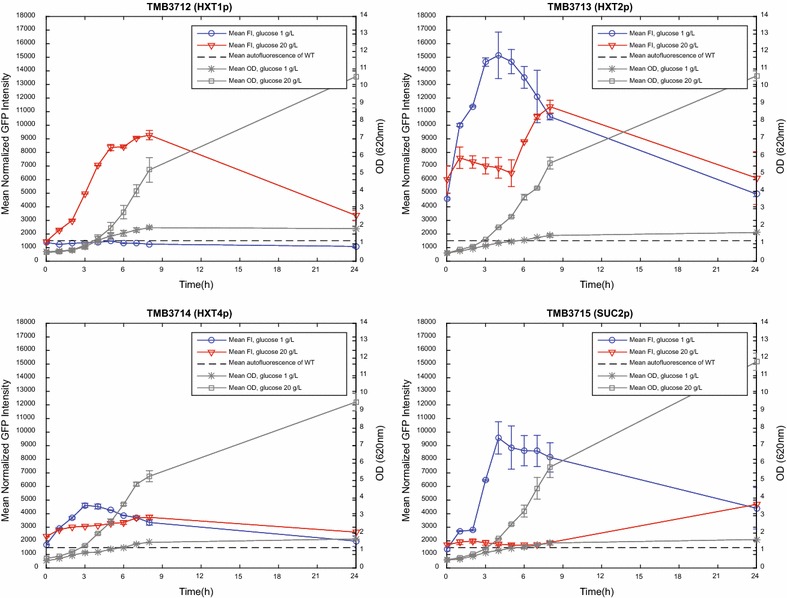
Evaluation of the induction/repression response of the TMB3712-3715 biosensor strains. Cultivations were performed in 1 and 20 g/L glucose (in YNB-KHPthalate medium) in order to enable validation against Table 2. *Error bars* for growth (OD) represent the standard deviation between two biological replicates, whereas the biological replicates for the FI are presented individually (*solid* and *dashed lines*)

**Fig. 3:**
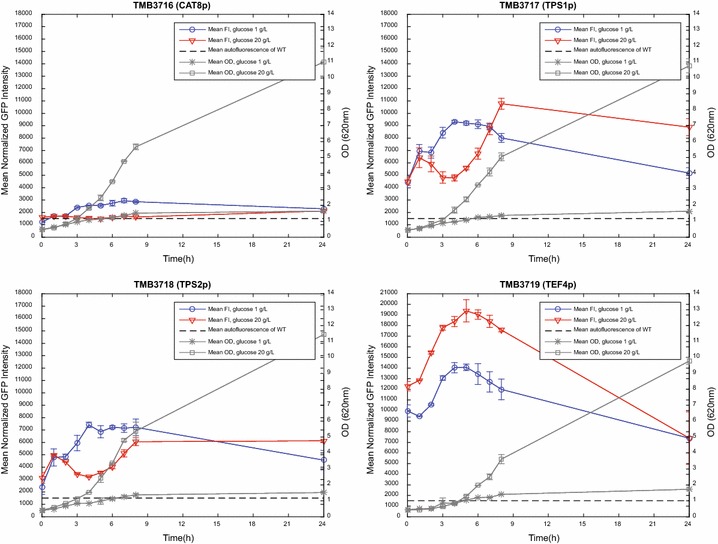
Evaluation of the induction/repression response of the TMB3716-3719 biosensor strains. As with Fig. 2, the cultivations were performed in 1 and 20 g/L glucose (in YNB-KHPthalate medium) in order to enable validation against Table 2. *Error bars* for growth (OD) represent the standard deviation between two biological replicates, whereas the biological replicates for the FI are presented individually (*solid* and *dashed lines*)

The negative control strain TMB3711 (no GFP-cassette) was used to identify the cellular auto-fluorescence at the GFP emission wavelength over time (Additional file 1: Figure S4) and was used to establish a mean, time-independent FI baseline for the biosensor strains (Figs. 2, 3). Also evident from Additional file 1: Figure S4, is that the (size normalized) auto-fluorescence does not change neither during true growth (glucose 20 g/L) nor during carbon starvation (glucose 1 g/L). To account for the fact that larger cells display a higher fluorescence than smaller ones, all FI signals were normalized to cell size using the Knijnenburg morphology correction model for Matlab [57], resulting in improved Gaussian distributions in the FI histograms. An example of the look of the FI histograms before and after normalization is available in Additional file 1: Figure S3.

By comparing the FI curves from the different conditions in Figs. 2, 3 with the known physiological outcomes for the corresponding strain and condition (listed in Table 2) it was found that the fluorescent response of the TMB3712-3718 biosensor constructs correlated with the reported induction/repression conditions from literature. This shows that GFP constructs such as these are indeed conceivable systems for monitoring the signaling state of the yeast cell. As was expected, the growth profiles (OD; Figs. 2, 3) and maximum specific growth rates (µ_max_; Additional file 1: Table S3) were similar between the nine strains, which corroborates that the physiology of these strains was unaffected by the integration of the biosensor cassettes. Furthermore, the metabolite profiles of extracellular glucose, glycerol, acetate and ethanol were also tantamount across the panel of strains (Additional file 1: Figure S5), showing that the integration of the sensor constructs did not interfere with the central metabolism. It was found that approximately 7 g/L remained in the 20 g/L glucose cultures after 8 h, which would still be high enough not to alter the induction/repression pattern (Table 2). Full carbon depletion occurred between 8 h and 24 h (Additional file 1: Figure S5).

TMB3719, however, proved to be very challenging to repress during the pre-culture, as this promoter (*TEF4p*) is known to be repressed by stress conditions (Table 2). Attempts were made with high glucose (40 g/L for 12 h) and respiratory conditions (Ethanol 3% v/v; 48 and 72 h) before settling on glucose 20 g/L for 24 h. Although this proved to be a more stressful, and thus more repressing, condition than in the other attempts, the sensor was still highly induced at 0 h (Fig. 3).

**Fig. 4:**
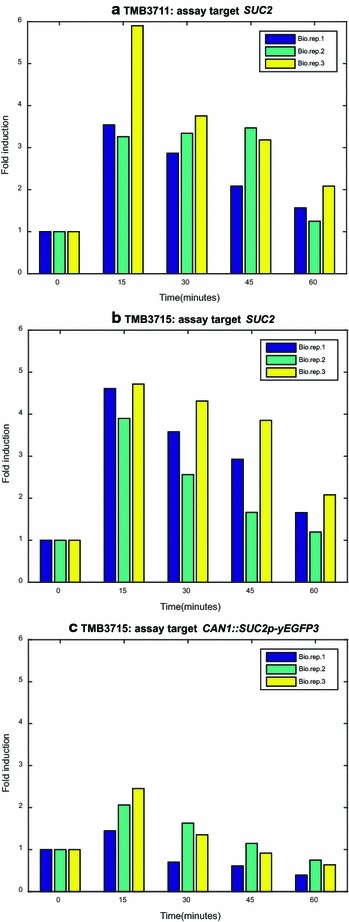
Results of the RT-qPCR assays. The fold induction of the expression of the endogenous *SUC2* gene during induction conditions (glucose 1 G/L) in **a** the negative control strain (TMB3711; no GFP), and **b** in the *SUC2* biosensor strain (TMB3715). **c** Fold induction of the *yEGFP3* gene in the biosensor construct (under control of the *SUC2* promoter, integrated in the *CAN1* locus)

To further validate that the observed GFP response indeed reflects the signaling patterns in these strains during high and low glucose conditions, and, more importantly, to ensure that the second promoter copy present in the genome after integration of the biosensor plasmids did not affect the endogenous expression, the transient mRNA levels were assessed with an RT-qPCR assay. Since the genetic construct, integration locus and plasmid copy number (double homologous integration) was identical for all the biosensor strains, except for the promoter region preceding the *yEGFP3* cassette, mRNA profile validation was performed for one biosensor strain (TMB3715; *SUC2p*) and the negative control strain (TMB3711). *SUC2* was chosen the since it encodes an enzyme (invertase) that can be easily assayed (which is not the case for many of the other biosensor genes, e.g. the hexose transporters); thus *SUC2* was used as a model to validate the biosensor construct versus the native gene on both the transcript and protein level—with respect to the integration locus of the cassette and the promoter copy number (one endogenous, one in the biosensor cassette). It was found that there was no significant difference in expression profile for the endogenous *SUC2* gene between the two strains and that the profile of the endogenous *SUC2* matched the one of the *SUC2p*-*yEGFP3* construct in TMB3715 (Fig. 4). This demonstrated that (1) the integration of the biosensor cassette did not alter the transcription phenotype despite there being two copies of the promoter in the genome (one endogenous, and one in the sensor construct; here: *SUC2p*), (2) that the observed (cumulative) increase in GFP signal (Fig. 2) was reciprocal to the transient GFP-transcript pulse (Fig. 4) and (3) that the chosen integration locus (*CAN1*) resulted in lower fold expression levels than the endogenous *SUC2* but that this did not obstruct the functionality of the sensor. It should be noted that though the behavior of *SUC2p* cannot be superimposed on that of the other promoters of this study, the correlation of Figs. 2, 3 with Table 2 have already given good indications to the functionality of all eight biosensors of this study.

Finally, to fully ascertain that the biosensor constructs truly were non-invasive, the phenotype of the *SUC2p*-*yEGFP3* strain was also assessed on the protein level by benchmarking the enzymatic activity of invertase (the protein product of *SUC2*) in TMB3715 to that of the negative control TMB3711. The endpoint invertase activity in cell extracts did not differ significantly between the two strains when cultivated in 1 g/l (inducing conditions for *SUC2*/invertase) (Additional file 1: Figure S6). Assay saturation (equivalent to 500 mM glucose without sucrose [62]) was reached in the samples from 2.5 to 3 h of cultivation, and therefore marked the end of the assay.

It should be noted that it is easy to follow the build-up of GFP in our biosensor system, but that the post-peak signal should preferably only be used for trends. This can be attributed to the facts that gene expression at that time will undergo a growth phase coupled shift [65] and that the fluorescent half-life of the yeast yEGFP3 protein is circa 7.5 h [66]. Alternative GFP cassettes with significantly lower half-life (34 min) exist [66], but is out of the question for the current study due to the invasive properties of the ATP-dependent degradation process [67] of the alternative GFP construct.

### Screening of the signaling response to xylose and other carbon sources

Once verified, the biosensor strains were used to screen for the signaling response on a panel of different carbon sources with a main focus on different combinations of xylose. To accomplish this, a protocol for microtiter plate screening was developed based on the Accuri C6 flow cytometer autosampler. Data analysis was again performed with our custom in-house Matlab and Python scripts and Knijenburg model [57] (cf. Methods section), producing a high-throughput in silico pipeline from data acquisition to post-data analysis. For each condition in this dataset, the fold change from the 0 h sample of the same condition was calculated in order to enable comparisons between conditions and to facilitate the overview of the microtiter plate data (Table 3; Additional file 1: Table S4).

**Table 3:** Results of the microtiter-plate screening of the biosensors strains on glucose, xylose and a co-culture thereof, given in terms of FI fold induction

Strain	Glucose 5 g/L^a^		Xylose 50 g/L + Gluc. 5 g/L		Xylose 50 g/L^b^	
	3 h	6 h	3 h	6 h	3 h	6 h
TMB3711 (No GFP)	1.00 ± 0.010	0.92 ± 0.056	1.04 ± 0.010*	1.02 ± 0.041 *	0.79 ± 0.017	0.78 ± 0.023
TMB3712 (*HXT1p*)	1.21 ± 0.016	1.23 ± 0.013	1.48 ± 0.011*	1.70 ± 0.011 *	0.83 ± 0.015	0.84 ± 0.010
TMB3713 (*HXT2p*)	1.10 ± 0.264	1.34 ± 0.11	1.37 ± 0.26	2.01 ± 0.55*	0.89 ± 0.039	0.89 ± 0.046
TMB3714 (*HXT4p*)	1.77 ± 0.058	2.47 ± 0.22	2.15 ± 0.31*	3.45 ± 0.62*	1.08 ± 0.25	1.31 ± 0.51
TMB3715 (*SUC2p*)	0.98 ± 0.15	3.51 ± 0.75	1.13 ± 0.10	5.15 ± 0.62*	0.95 ± 0.12	1.06 ± 0.21
TMB3716 (*CAT8p*)	0.98 ± 0.002	1.14 ± 0.039	1.08 ± 0.037*	1.13 ± 0.037	0.82 ± 0.003	0.84 ± 0.018
TMB3717 (*TPS1p*)	0.74 ± 0.051	1.23 ± 0.075	0.70 ± 0.048	0.77 ± 0.047*	0.92 ± 0.006	0.91 ± 0.029
TMB3718 (*TPS2p*)	0.85 ± 0.026	1.61 ± 0.084	0.95 ± 0.076*	1.05 ± 0.032*	0.85 ± 0.012	0.85 ± 0.026
TMB3719 (*TEF4p*)	1.35 ± 0.013	1.46 ± 0.010	1.28 ± 0.018*	1.42 ± 0.0032	0.87 ± 0.012	0.96 ± 0.010

The FI signal was normalized to the corresponding 0 h signal of the given condition and strain. A value of 1 corresponds to repression (i.e. no fold change since time 0 h). A one-way ANOVA with a multiple comparison test was performed to statistically compare the results from the different conditions

* Significantly different from the glucose 5 g/L fold change from the same hour (one-way ANOVA with a multiple comparison test; p ≤ 0.05)

^a^Glucose 5 g/L was significantly different from the xylose 50 g/L in all strains and times except for 3 h for TMB3713, TMB3715, TMB3718 and 6 h for TMB3713

^b^Xylose 50 g/L was significantly different from the xylose-glucose co-culture in all strains and times except for TMB3715 at 3 h

Glucose 1 g/L and 20 g/L displayed the same trend in the microtiter plates as in the previous shake flask cultivations (calculated as the fold change from the 0 h baseline; Additional file 1: Figure S7). This demonstrates that the scale-down to microtiter plate cultures did not affect the biosensor signal, while conserving the reproducibility of the assay. It can however be noted that the microplate 0 h-measurement of the *TPS1p/2p* sensors rather seem to reflect the 1 h-point of the shake flask cultures; due to the behavior of the FI pattern in the 1–6 h interval for these two sensors (Fig. 3), a different fold change response direction was therefore found in the microtiter plate experiment compared to the shake flasks (Additional file 1: Figure S7).

Xylose has in previous studies commonly been supplied to engineered *S. cerevisiae* strains in high concentration (50 g/L) in order to improve the uptake rate, as only unspecific pentose transporters exists in this species [12, 68]. Consequently, 50 g/L xylose was used to screen the strains for any signaling response to this pentose sugar. Xylose did not elicit any significant response in any of the strains (Table 3) and the fold change at 3 and 6 h (compared to the signal at 0 h) was comparable to true carbon starvation as was assessed using YNB-KHPthalate medium without any added carbon source (Additional file 1: Table S4).

It has previously been observed that the xylose uptake can be improved by co-substrate cultures with low concentrations of glucose [69]. In order for the glucose to quickly be consumed, but not start out low enough to induce the biosensors strains known to be induced by 1 g/L glucose (Table 2), a xylose (50 g/L) and glucose (5 g/L) co-culture was evaluated, as well as glucose 5 g/L alone in order to be able to distinguish between pentose and hexose effect. It was found that for the biosensors based on hexose transporter promoters (TMB3712-3714), this co-substrate cultivation resulted in a slightly higher fold-change than glucose 5 g/L alone (Table 3). In fact, it was found that the xylose–glucose co-culture was significantly different from the glucose 5 g/L results for 13 out of the 18 measured time points in Table 3, showing that the higher fold change in the co-substrate cultures was not an effect of the glucose alone, but of the presence of both sugars (Table 3). The xylose that was used in the study had a reported Lot purity of 99.7%, and it is therefore unlikely that the response from the high-affinity transporters is caused by low levels of contaminating sugars. To confirm this, the xylose stock solution that was used throughout this study was analyzed by HPLC, and no other peaks than the expected xylose peak were found (Limit-of-detection: 0.8 g/L).

On the same note, co-substrate cultures of xylose (50 g/L) and glycerol (3% v/v) was used to assess the effect of xylose during respiratory growth. Since it has been hypothesized that xylose exhibits a “non-fermentable carbon source”—response in *S. cerevisiae* [13, 14, 17], evaluation of the sugar signaling during respiratory conditions was performed. However, neither glycerol alone nor in co-culture with xylose, resulted in any major induction fold change throughout the course of the screening (Additional file 1: Table S4).

**Fig. 5:**
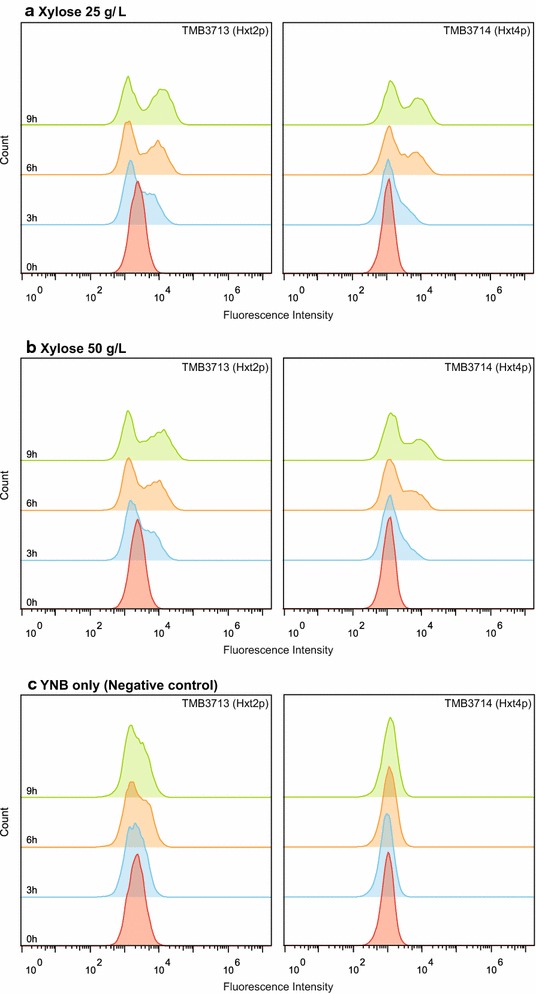
Overlay histogram plots from the raw (non-normalized) data of TMB3713 (*HXT2p*) and TMB3714 (*HXT4p*). Graphs show the distribution of the Fluorescence Intensity per registered event in the sample at each of the four time points (0, 3, 6 and 9 h). The Gaussian distribution seen at time 0 h for all strains signified homogenous populations, whereas deviations from the normal distribution implied population heterogeneities. The strains were cultivated in xylose 25 and 50 g/L and YNB-KHPthalate medium without any carbon source. It evident from **a** and **b** that two subpopulations appear from 3 h and forward, and that this is not the case in the negative control (YNB only). The left subpopulations are equivalent to the cellular autofluorescence (cf. **c**), whereas the right subpopulations are clearly induced

In order to determine if xylose could have any dose-dependent effect on the cellular signaling, all strains were also evaluated in microtiter plates with xylose concentrations ranging from 25 up to 100 g/L. The analysis time was also extended from 6 h to 9 h to fully assure that any possible lag in the sensor signal would not interfere with the results. For most reporter genes the fluorescence intensity fold-changes again showed unremarkable patterns (Additional file 1: Table S5); the *HXT* sensors, however, consistently displayed higher fold change levels than the remaining biosensors. Furthermore, a study of the raw, non-normalized fluorescence data, revealed that while TMB3712 (*HXT1p*) only showed one, well-defined, fluorescence population (Additional file 1: Figure S8), strains TMB3713 and TMB3714 (*HXT2p* and *HXT4p*, respectively) showed two distinct fluorescence subpopulations (Fig. 5). These two subpopulations were neither visible at the start of the experiment, nor did they appear for cultivations in YNB-KHPthalate only.

In order to evaluate the potential induction characteristics of the two populations, the FI histograms were manually gated and geometric means were calculated for the lower and higher intensity populations, respectively. The intensity values were then compared to the mean intensity of the starting population at 0 h (Table 4). These estimations, along with Fig. 5, made it evident that the percentage of fluorescence events belonging the higher intensity population increased over time and that this percentage was slightly higher for TMB3713 (*HXT2p*) independent on sampling point (Table 4). After 3 h in xylose 25 g/L the higher intensity populations for TMB3713 and TMB3714 had reached fold-changes of 2.69 ± 0.10 and 4.40 ± 0.10 respectively (Table 4), which was comparable to the induction achieved by glucose 1 g/L during the same time period (3.62 ± 0.130 and 4.27 ± 0.50, respectively; Additional file 1: Table S4).

**Table 4:** Population heterogeneities for the hexose transporter-based biosensors during xylose cultivations

Strain	Population	FI fold changes (25 g/L xylose)			FI fold changes (50 g/L xylose)		
		3 h	6 h	9 h	3 h	6 h	9 h
TMB3712 (*HXT1p*)	Single	1.24 ± 0.17	1.10 ± 0.10	1.00 ± 0.32	1.26 ± 0.36	1.23 ± 0.26	1.13 ± 0.31
	% of total population	100	100	100	100	100	100
TMB3713 (*HXT2p*)	High FI	2.69 ± 0.10	3.90 ± 0.28	5.16 ± 0.060	3.23 ± 0.21	3.70 ± 0.020	4.52 ± 0.082
	% of total population	36 ± 0.1	43 ± 6.1	51 ± 2.1	32 ± 4.1	42 ± 0.5	49 ± 2.1
	Low FI	0.66 ± 0.004	0.58 ± 0.031	0.58 ± 0.004	0.71 ± 0.018	0.60 ± 0.017	0.58 ± 0.030
	% of total	64 ± 0.1	57 ± 6.1	49 ± 2.1	68 ± 4.1	58 ± 0.5	51 ± 2.1
TMB3714 (*HXT4p*)	High FI	4.40 ± 0.10	6.39 ± 0.15	7.76 ± 0.10	5.29 ± 1.22	6.01 ± 0.26	8.14 ± 0.024
	% of total population	19 ± 3.3	37 ± 3.2	42 ± 2.7	13 ± 8.3	29 ± 1.8	35 ± 0.2
	Low FI	1.02 ± 0.004	1.04 ± 0.007	1.21 ± 0.012	1.11 ± 0.11	1.07 ± 0.047	1.15 ± 0.068
	% of total population	8.1 ± 3.3	63 ± 3.2	58 ± 2.7	87 ± 8.3	71 ± 1.8	65 ± 0.2

Peak fold changes and population distributions (percent of total cell population) during cultivation in 25 and 50 g/L xylose in non-normalized data (for the normalized data from this experiment, cf. Additional file 1: Table S5). TMB3712 (*HXT1p*) displayed an increase in FI after already 3 h, but the analyzed samples were consistently distributed in a single peak population. TMB3713 (*HXT2p*) and TMB3714 (*HXT4p*) were highly heterogeneous on the FL-1 (GFP) channel with two distinct peaks (one with low and one with high FI). The histograms of TMB3713-3714 are also displayed in Fig. 5. The experiments were performed in biological and technical duplicates

## Discussion

### Extracellular xylose is not in itself sensed by *S. cerevisiae*

Over the years, transcriptomics, metabolomics and metabolic flux analysis studies have uncovered the unusual cellular response that xylose assimilation triggers in *S. cerevisiae*. Several significant differences between xylose and glucose on the regulation of the central carbon metabolism have been highlighted in the past, including: catabolite repression patterns [70], respiratory metabolism during oxygen limited cultivations with xylose as the sole carbon source (as opposed to the expected respiro-fermentative metabolism) [19]; expression of respiratory pathway genes on xylose during anaerobiosis [13, 71]; a decrease in the concentration of glycolysis- and pentose phosphate pathway-related precursor metabolites when shifting from glucose to xylose [17]; and an accumulation of aromatic amino acids in yeast cultivated on xylose, that was comparable to the response of starving cells [18, 72]. Taken together, these results have hinted towards a major issue in the xylose sensing and recognition of *S. cerevisiae*, and we believe that this issue is likely to be a cause of the current bottleneck(s) hindering efficient yeast valorization of lignocellulosic material.

The current study has indicated that endogenous mechanisms for xylose signaling do exist in *S. cerevisiae* and that this signal does not seem to originate from sensing of extracellular xylose. Most of our biosensors remained unresponsive to xylose, regardless of it being presented to the cell as a sole carbon source or together with other, fermentable or non-fermentable, carbon sources. These findings would indicate that, for the majority of its carbon sensing pathways, *wild type* non-xylose utilizing *S. cerevisiae* cannot sense extracellular xylose, but would rather sense the lack of fermentable carbon sources (previously suggested in e.g. [16]). However, as will be discussed below, we hypothesize that this is not necessarily the fact for *internalized* xylose.

### Flow cytometry illuminates otherwise unseen population heterogeneities on xylose

A key feature of the chosen methodology is that is allows for the assessment of sugar sensing population heterogeneities through the means of flow cytometry. Since all strains (and therefore all cells in each measured population) have been engineered to have one single copy of the biosensor, any occurrence of subpopulations can be considered to be of true physiological relevance. When a consistently higher mean FI was discovered for the *HXT1/2/4* biosensors in the xylose titer dataset (Additional file 1: Table S5), we traced the effect down to the population distribution level; this allowed us to identify subpopulations expressing significantly higher amounts of GFP in non-growing TMB3713 (*HXT2p*) and TMB3714 (*HXT4p*) incubated with 25–100 g/L xylose (Fig. 5; Table 4 highlights the results of the 25 and 50 g/L cultivations). This heterogeneous population distribution on the FI channel would have gone undetected with conventional fluorimetry or transcriptomics, where only population averages are considered (i.e. methods that will detect changes in average signal, but cannot identify subpopulations). In both the TMB3713 and TMB3714 strains, the higher of the two FI populations showed FI fold-changes (Fig. 5; Table 4) comparable to the induction patterns found for 1 g/L glucose (Additional file 1: Table S4), the condition considered to yield the highest induction for these gene targets. Despite the slightly higher mean FI of TMB3712 (*HXT1p;* Additional file 1: Table S5), this strain did not display any subpopulations during these conditions (Additional file 1: Figure S8).

The presence of these fluorescent subpopulations allowed us to surmise a few of the characteristics of this apparent xylose sensing effect. We hypothesize that this sensing requires xylose to be transported into the cell. Out of the two sensors controlling *HXT1/2/4* induction, Snf3p and Rgt2p, the former has been reported to be glucose repressed while the latter is expressed at low levels regardless of glucose or xylose concentrations [14, 20, 73], indicating that during incubation with xylose all cells would have the same probability of sensing the extracellular xylose at any specific time point. Were the xylose sensing only contingent on recognition at the cell membrane, all cells would be equally susceptible to induction and the fluorescence profiles would remain single-population, but this is not the case in the current results for TMB3713-3714. Another factor weighing into this conclusion is that none of the Snf3p/Rgt2p pathway regulated genes showed any split FI-histogram populations under glucose conditions (data not shown). We therefore hypothesize that at least one component of the natural Snf3p/Rgt2p signaling pathway is missing for xylose, and this missing piece is likely to be the system for extracellular recognition.

During the pre-cultivations, the biosensor strains were grown in conditions that were repressing towards their particular gene target. In the case of TMB3713 (*HXT2p*) and TMB3714 (*HXT4p*), a high glucose content (40 g/L for 12 h) was used as the repressing condition. While this condition will repress the high-affinity hexose transporters, low-affinity transporters such as *HXT1* will be in an induced stage at the end of the pre-culture/start of the xylose incubation. Although these low-affinity transporters have been reported to have an affinity for xylose so low that they cannot support growth of otherwise xylose-utilizing yeast strains [74], it is known that they do have some transport capacity for this pentose [75]. It is therefore possible that the Hxt1p membrane transport protein, or any of the other glucose induced transporter proteins, are present on the cell membrane when the yeast cells are transferred from the glucose 40 g/L pre-culture to the xylose-containing media. From here—and this is the core of the present hypothesis—a stochastic event decides whether or not the cell will take up the xylose (via the expressed hexose transporters). For the subpopulation in which this event occurred, the internalized xylose molecules were then able to induce/derepress the *HXT2/4* genes, as was demonstrated by the population distributions observed in Fig. 5.

Partial derepression of high-affinity hexose transporter genes for *S. cerevisiae* was also found in a transcriptomics study by Salusjarvi et al. [14], comparing expression profiles while metabolizing xylose or in different states of catabolite repression. Although the authors also suggest a potential xylose effect on the Snf3/Rgt2 pathway, their study cannot differentiate between sensing of extracellular or intracellular xylose, especially since xylose was confirmed to be taken up and metabolized. In contrast, our study focused on the inherent effect of xylose—and not its metabolites—on native yeast, as well as elucidating the population heterogeneities obscured by transcriptomic approaches.

## Conclusions

Through this study, we have generated and validated a panel of in vivo biosensors that allows for rapid assessment of the sugar signaling state of the *S. cerevisiae* cell. Furthermore, this study has, to our knowledge, demonstrated that extracellular xylose itself does not trigger a regulatory response, as the current results imply that the previously reported respiratory response on xylose is due to the lack of glucose and is not an effect of the presence of extracellular xylose. Furthermore, the results of the current study indicate for the first time a cellular mechanism for recognition of internalized xylose in *S. cerevisiae*; however, future work dedicated to this hypothesis is required in order to fully ascertain this. Accordingly, this creates a strong impetus for metabolic engineering of the sugar signaling pathways as the next logical step in order to improve xylose utilization and valorization in this yeast.

## Additional file

**Additional file 1.** Additional materials and methods, figures and tables.

## Abbreviations

FI: fluorescence intensity
GFP: green fluorescent protein
ORF: open reading frame
YNB: yeast nitrogen base
