# Supplementary material for: Real-time monitoring of the sugar sensing in Saccharomyces cerevisiae indicates endogenous mechanisms for xylose signaling
Source: Microb Cell Fact. 2016 Oct 24;15:183. doi: 10.1186/s12934-016-0580-x (PMC5078928; doi:10.1186/s12934-016-0580-x)
Supplement: Supplementary file 1 — Additional file 1. Additional materials and methods, figures and tables. [file 12934_2016_580_MOESM1_ESM.pdf]

Supporting Information for:

Real-time monitoring of the sugar sensing in *Saccharomyces cerevisiae* indicates endogenous mechanisms for xylose signaling

Daniel P Brink[†]*, Celina Borgström[†], Felipe G Tueros and Marie F Gorwa-Grauslund

Applied Microbiology, Department of Chemistry, Lund University, Lund, Sweden

*Corresponding author

E- mail: daniel.brink@tmb.lth.se

[†]These authors contributed equally to this work

Supplementary Materials and Methods

High-throughput cell size normalization script for flow cytometry

Flow cytometry, not unlike genome sequencing and RNA-seq, generates *Big Data* due to its single cell analysis over multiple channels. This calls for novel *in silico* tools for facilitating post-data processing, especially in experimental designs where the number of runs, replicates and conditions start to increase. We have designed Matlab (Release R2015a, The MathWorks, Inc., Natick, MA, US) and Python (v3, The Python Software Foundation, US) scripts that within minutes processes raw .fcs-files (here outputted by the BD Accuri flow cytometer; Becton-Dickinson, NJ, US), normalizes the fluorescence intensity (FI) on channel FL1-H to cell size by calling the Knijnenburg morphology normalization model [1], and plots the geometrical mean of each normalized histogram versus time as either scatter or bar plots. Thus we have generated a high-throughput pipeline from data acquisition to post-processing that significantly decreases the hands-on analysis time. Since there is no set limitation in the number of strains, replicates and conditions that can be assess with these custom scripts, they are applicable both to batch and microtiter plate cultivation data.

The main script that needs to be called in order to run the pipe line is the *FI_size_normalization_loader.m* (Matlab). Users will have to download the Knijnenburg model separately according to the author's instructions [1] and store *FI_size_normalization_loader.m* in the root of the Knijnenburg model folder. Our custom Matlab script recognises .fcs-files stored in the following folder hierarchy: */<strain>/<biological replicate>/<conditions>*. Filenames must include the sample time (in hours) and the folder names are used to keep track of the files during the processing. In order to facilitate the control of filenames and folder hierarchy, a custom Python script (v3, The Python Software Foundation, US) was also written.

The *batch_name_change_accuri_fc_data.py* script was specifically designed for use with .fcs-files output by the Accuri software. A specific feature of this software is that it adds the acquisition position of the sample to each sample with exported as .fcs: e.g. A01 TMB3711 1g.L 0h. The Python script

uses the position in the acquisition matrix (rows A-H, columns 01-12) to rename and sort the files in the required hierarchy. The user inputs the desired names in the *accuri_data_matrix.txt* template and when run the script outputs data that can directly be used as in-data for *FI_size_normalization_loader.m* script in Matlab. Although it is however possible to rename the .fcs files manually (as long as the file names end with Xh.fcs, where X is the sample time), we recommend users to use the *batch_name_change_accuri_fc_data.py-FI_size_normalization_loader.m* pipeline for convenience.

The *FI_size_normalization_loader.m* comes with a number of options to customize the output of the processed data. The user will have to choose if they want scatter plots (`styler='time'`) or bar plots (`styler='bar'`). Optional settings are: plot each replicate individually (`err='0'`; default) or as means with standard deviation errorbars (`err='1'`); and to show the results of the normalized histograms (`norm_plot='1'`) or not (`norm_plot='0'; default`). For more information on the scripts, please see the readme-file in the script archive.

At the moment, the script can only normalize and plot data from one channel of the flow cytometer as this was the desired feature for the current study. Users are welcome to modify the scripts to fit with their own projects as along as the current study is cited. An archive containing all custom scripts developed for the current study is available in Supplementary File S2 and has also been deposited on Github (https://github.com/tmbyeast/Flow-cytometry-tools). Possible future updates to the customs scripts will be stored on the Github page.

Adapted invertase assay

The invertase assay used in the current study was adapted from three previous protocols in order to generate a protocol for assessment of cell extracts that did not rely on the commonly used, but highly carcinogenic, o-dianisidine chromophore, but instead on the non-carcinogenic 4-aminoantipyrine/phenol reaction [2-4]. The assay is based on three coupled enzymatic reactions:

invertase in cell extracts converts added sucrose into fructose and glucose, followed by oxidation of the latter by glucose oxidase, producing D-glucono-1,5-lactone and hydrogen peroxide. In the third step hydrogen peroxide is used by horseradish peroxidase to produce a colored chromophore from an added substrate [3].

Cells were grown in 100 mL shake flask cultures with 1 g/L glucose as inducing condition (preceded by pre-pre- and pre-cultures as described in the *Cultivation conditions* section in the main publication). Samples of 5 mL were harvested by centrifugation (1800 RCF, 5 min at room temperature), washed once in 5 mL sterile water prior to supernatant removal with an FTA-1 aspirator (Biosan, Riga, Latvia). Cell pellets were stored in -80°C and all samples were kept on ice during subsequent cell lysis and sample pre-processing.

For the enzyme assay, the frozen cell pellets were thawed on ice and resuspended in 600 µL 0.1 M $NaC_2H_3O_2$ (Na-Ac). Lysis of cells was done by glass bead beating in a Precellys 24 (Bertin Technologies, France; 5000 rpm, 3 cycles, 45s/cycle, 30 s pause between cycles) cooled by a Cryolus temperature controller (Bertin Technologies, France) with dry-ice. 400 µL of the supernatant was transferred to a fresh 1.5 mL tube and centrifuged at 500 RCF and 0°C for 5 minutes [4]. The supernatant was then transferred to 150 µL of 0.1 M Na-Ac, generating the final product of the sample pre-processing. 5 µl of this solution was used for the invertase assay by adding it to 100 µL fresh 0.1 M Na-Ac [4].

The first assay step (using the invertase in the cell extracts) was started by adding 25 µL freshly prepared 0.5 M sucrose to the assay tubes and incubating in a 37°C water bath for 20 minutes [3]. Four controls were prepared for each assay: I) cells without sucrose, II) sucrose without cells, III) 250 mM glucose and IV) 500 mM glucose [3]. The invertase reaction was stopped by addition of 150 µL 0.2 M K_2HPO_4 and boiling for 3 minutes in a heat block [3].

To start the second and third assay steps (glucose oxidase and peroxidase), 0.5 mL 4-aminoantipyrine-based coloring reagent solution (0.1 M K_2HPO_4, pH 7; 2.5 mg/mL glucose oxidase (>15,000 U/g; Sigma-Aldrich, St. Louis, MO, USA); 0.5 mg/ml Type II horseradish peroxidase (200 U/mg; Sigma-Aldrich, St. Louis, MO, USA) [4]; 1.48 mM 4-aminoantipyrine; 21.25 mM phenol [2]; wrapped in foil and stored at 4°C) was added to the tubes that were then incubated in a 37°C water bath for an additional 20 minutes. Finally, absorbance was measured at 540nm in technical duplicates using a Ultrospec 2100 Pro spectrophotometer (Amersham Biosciences, Uppsala, Sweden).

Total protein content in the cell extracts samples were quantified using the Coomassie (Bradford) Protein Assay Kit (Thermo Scientific; Waltham, MA, US) with 5 µL of each cell extract sample. The assay outcome was determined with a Multiscan Ascent microtiter plate reader (Thermo Scientific; Waltham, MA, US) at 595nm.

The end-point invertase concentration for every sample was calculated using Lambert-Beer's law and the molar absorptivity 13,900 $M^{-1}cm^{-1}$ for the resulting product of the 4-aminoantipyrine/phenol assay (4-N-(1,4-benzoquinoneimine)-antipyrine) [5]. The specific invertase activity was then determined by dividing the invertase concentration with the corresponding total protein content and invertase reaction time (20 min), yielding the unit µmol μg^{-1} min^{-1}.

Supplementary Figures

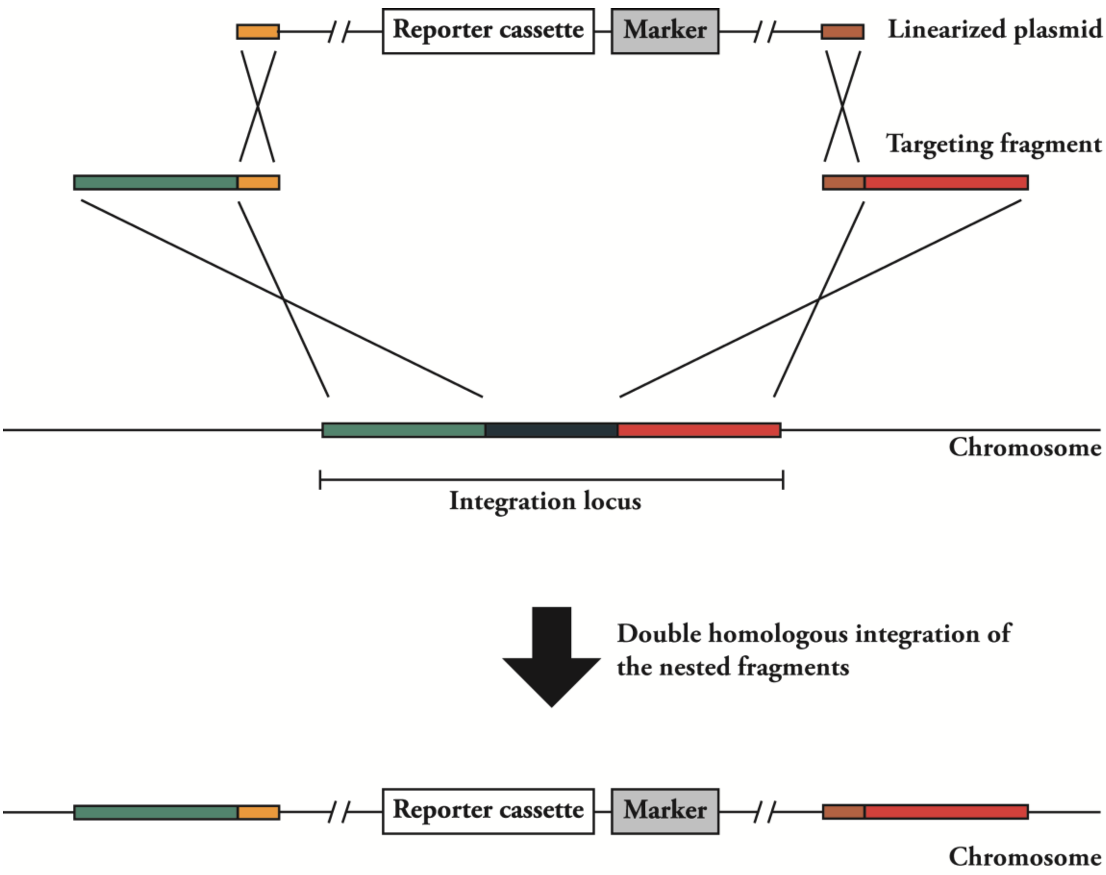

Figure S1. Schematic representation of the utilized strategy for double homologous, single-copy integration of the GFP-reporter plasmids in the *S. cerevisiae* genome. Correct integration of the linearized plasmid was aided by targeting fragments with homology to both the plasmid and the integration locus. Colors represent homologous regions.

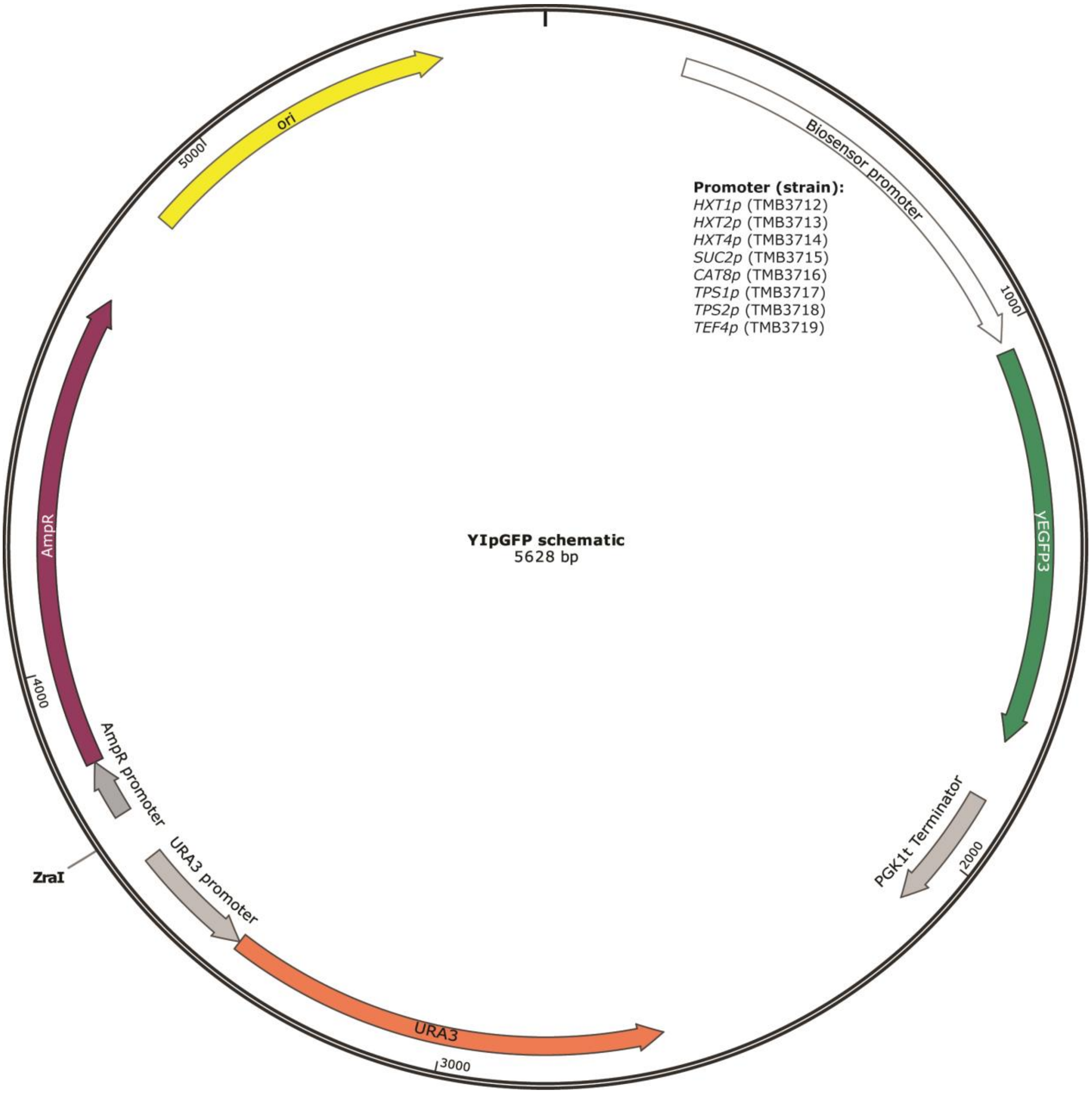

Figure S2. Schematic illustration of the biosensor plasmids. The biosensor promoter cassette was varied between the eight biosensor plasmids (see note next to cassette). *ZraI* restriction enzyme was used linearize the plasmid prior to yeast integration.

A

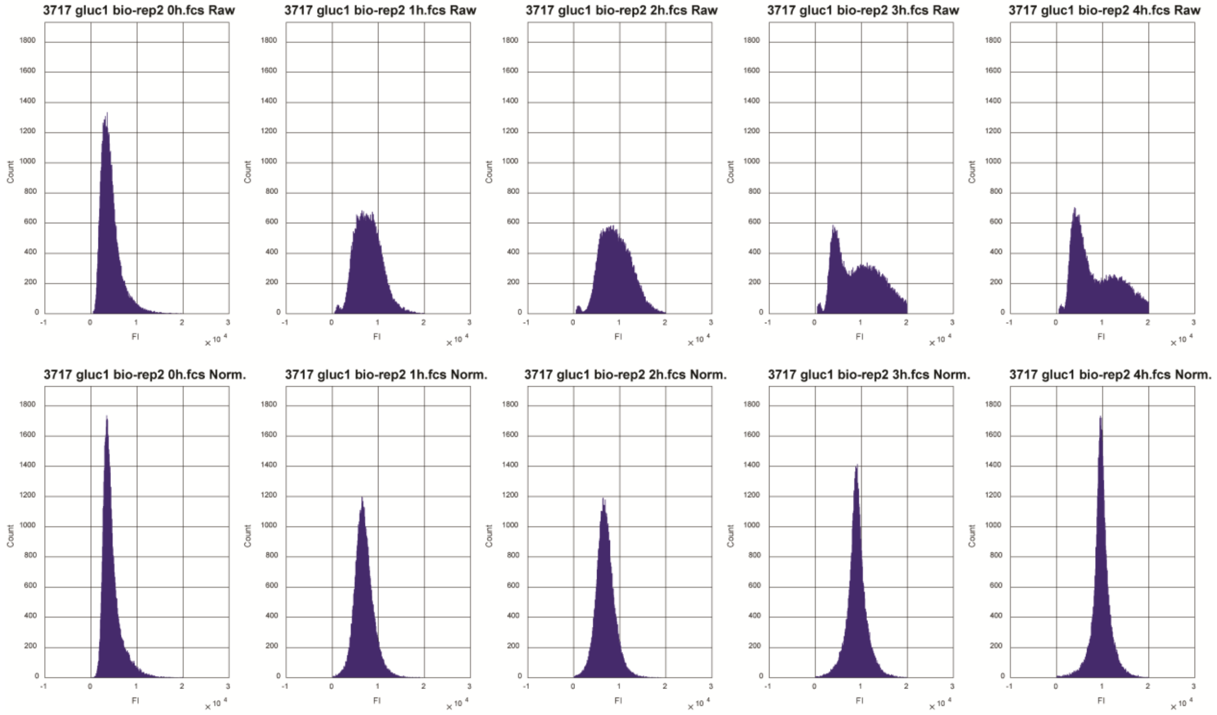
3717 gluc1 bio-rep2 0h.fcs Raw
3717 gluc1 bio-rep2 1h.fcs Raw
3717 gluc1 bio-rep2 2h.fcs Raw
3717 gluc1 bio-rep2 3h.fcs Raw
3717 gluc1 bio-rep2 4h.fcs Raw
3717 gluc1 bio-rep2 0h.fcs Norm.
3717 gluc1 bio-rep2 1h.fcs Norm.
3717 gluc1 bio-rep2 2h.fcs Norm.
3717 gluc1 bio-rep2 3h.fcs Norm.
3717 gluc1 bio-rep2 4h.fcs Norm.
Count
FI

B

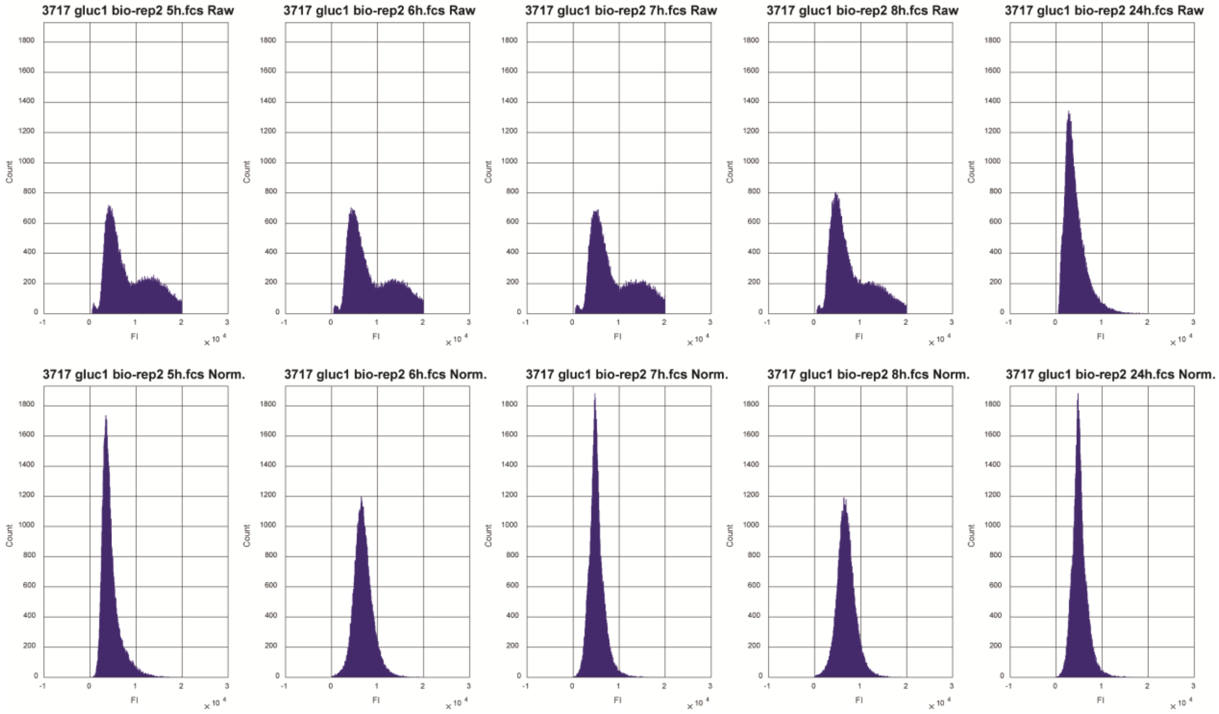
3717 gluc1 bio-rep2 5h.fcs Raw
3717 gluc1 bio-rep2 6h.fcs Raw
3717 gluc1 bio-rep2 7h.fcs Raw
3717 gluc1 bio-rep2 8h.fcs Raw
3717 gluc1 bio-rep2 24h.fcs Raw
3717 gluc1 bio-rep2 5h.fcs Norm.
3717 gluc1 bio-rep2 6h.fcs Norm.
3717 gluc1 bio-rep2 7h.fcs Norm.
3717 gluc1 bio-rep2 8h.fcs Norm.
3717 gluc1 bio-rep2 24h.fcs Norm.
Count
FI

Figure S3. Example of the look of the FI histograms before and after normalization. The second biological replicate of strain TMB3717 cultivated with glucose 1g/L is here used as an example. The X-axes of the histograms represent the FI signal for GFP and the Y-axes the counts. A: results for 0-4h. B: results for 5-24h. Bottom rows of A and B show the normalized data from the corresponding raw data in the row above.

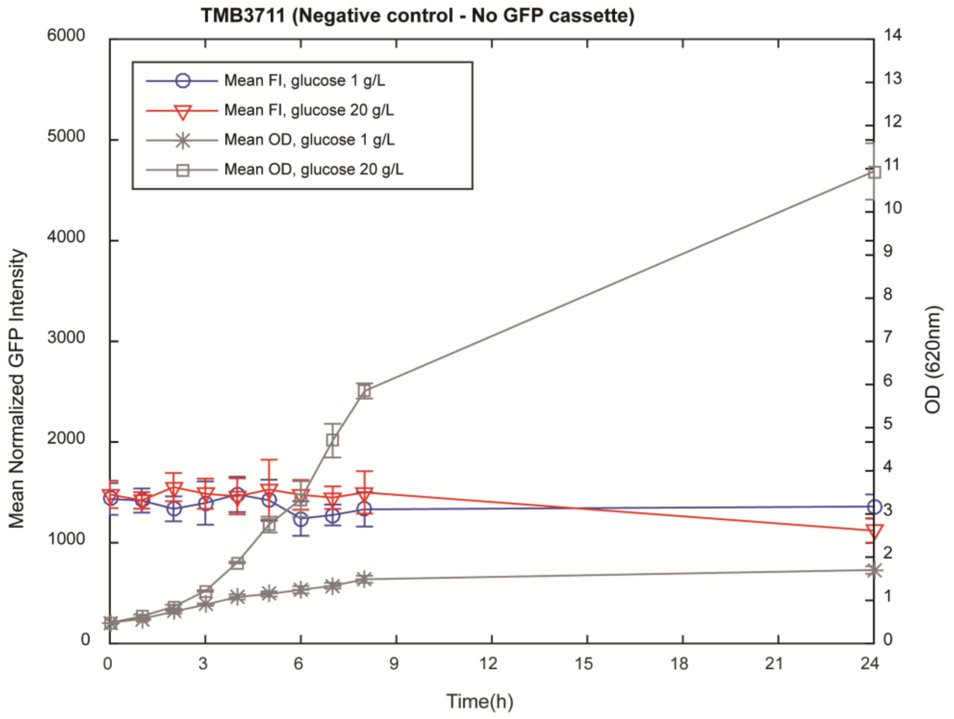

Figure S4. Evaluation of strain TMB3711 (negative control strain without the *yEGFP3* cassette) in terms of growth (OD) and fluorescence intensity (FI). Cultivations were performed in 1 g/L and 20 g/L glucose (YNB-KHPthalate medium) in two biological replicates. Due to the lack of the *GFP* gene in this strain, the above FI represents the autofluorescence of the TMB371X biosensor strains at the current excitation wavelength (488 nm).

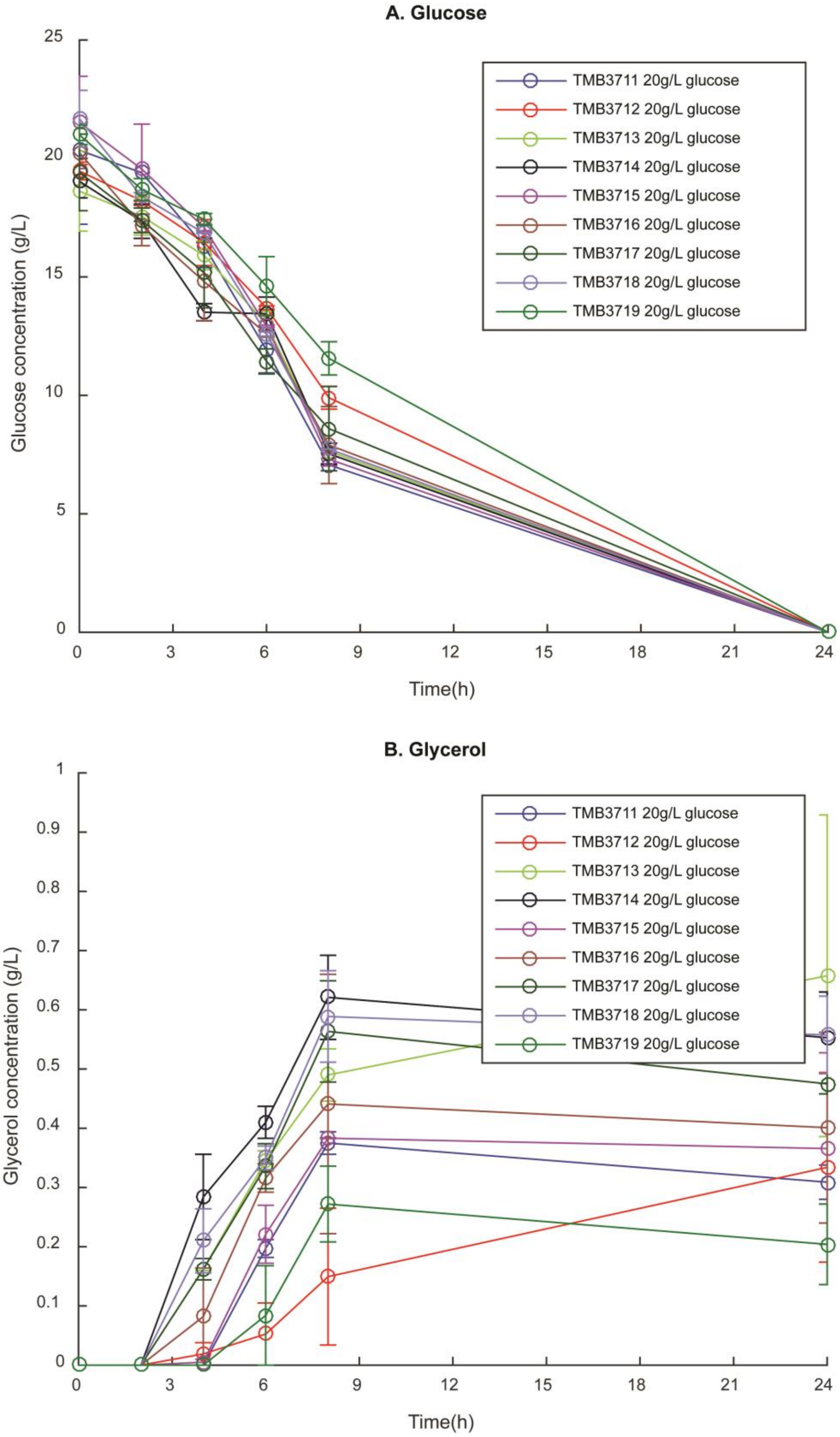
A. Glucose
Glucose concentration (g/L)
Time(h)
TMB3711 20g/L glucose
TMB3712 20g/L glucose
TMB3713 20g/L glucose
TMB3714 20g/L glucose
TMB3715 20g/L glucose
TMB3716 20g/L glucose
TMB3717 20g/L glucose
TMB3718 20g/L glucose
TMB3719 20g/L glucose
B. Glycerol
Glycerol concentration (g/L)
Time(h)
TMB3711 20g/L glucose
TMB3712 20g/L glucose
TMB3713 20g/L glucose
TMB3714 20g/L glucose
TMB3715 20g/L glucose
TMB3716 20g/L glucose
TMB3717 20g/L glucose
TMB3718 20g/L glucose
TMB3719 20g/L glucose

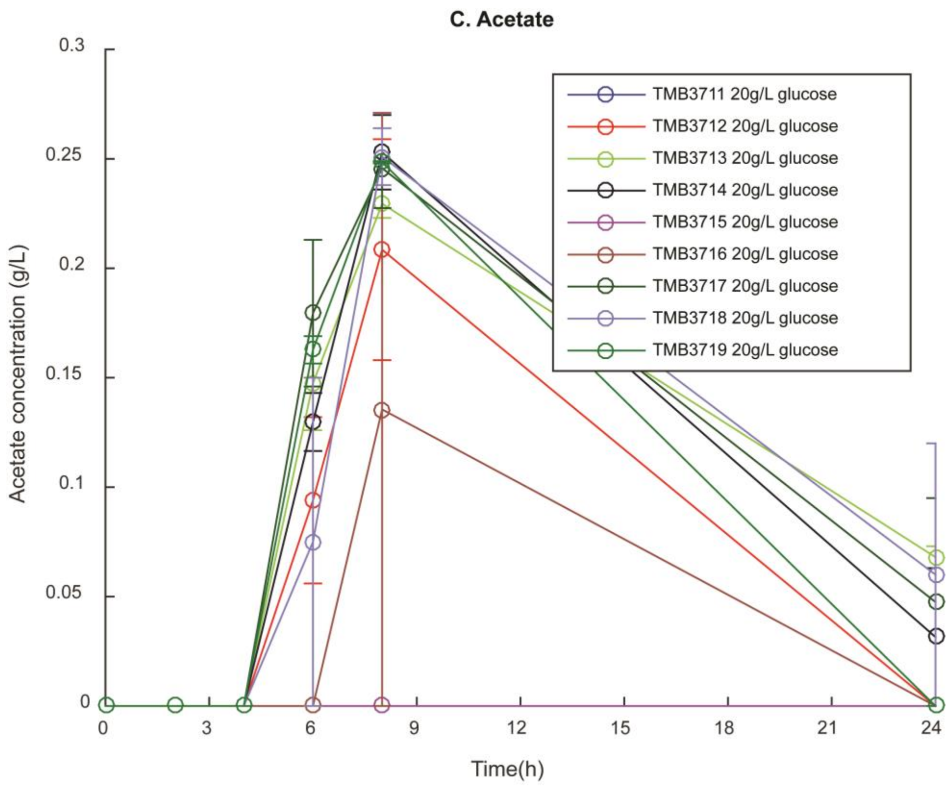

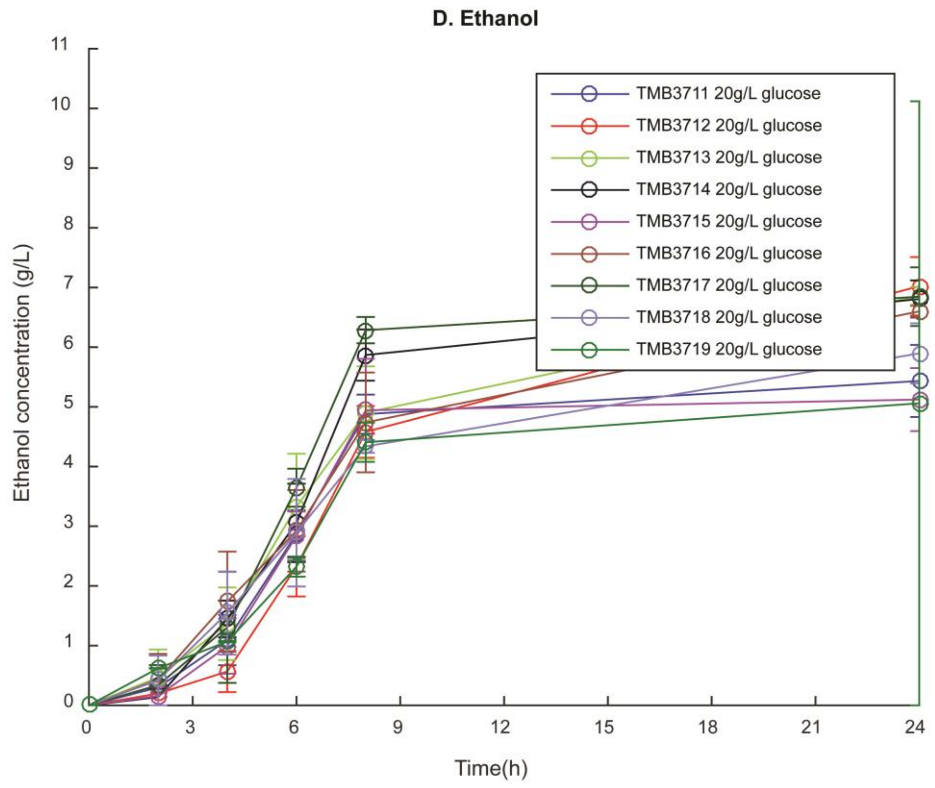

Figure S5. Metabolite profiles of the 24h 100 mL batch cultivations of the biosensor strains, as determined by HPLC. Acetate peaks were observed in all strains but could not always be quantified as some peaks were below the limit of detection of the standard curve (<0.05 g/L). The analysis was performed in two biological and two technical replicates.

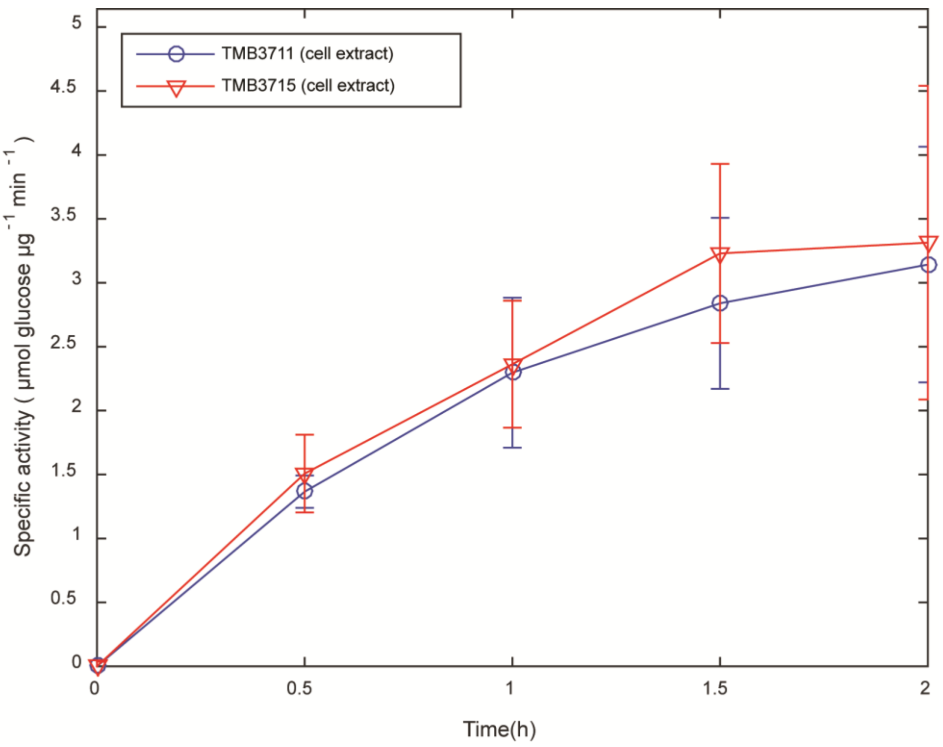

Figure S6. Invertase assay results. Specific invertase activity in cell extracts from TMB3711 (negative control) and TMB3715 (*SUC2p-yEGFP3* biosensor strain). The assay was performed in biological triplicates and the vertical bars indicate the standard deviation between the replicates.

A.

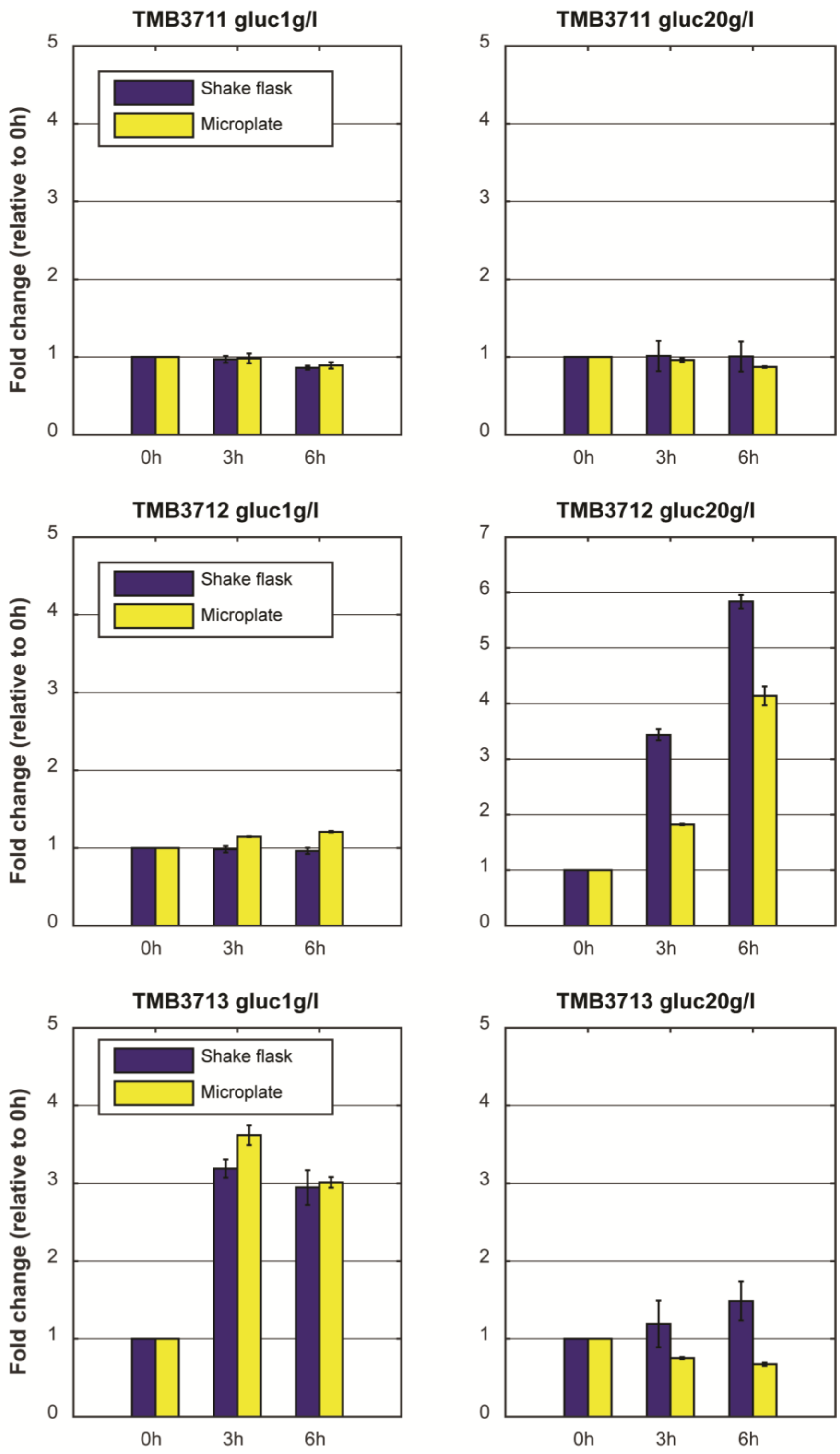
TMB3711 gluc1g/l
TMB3711 gluc20g/l
TMB3712 gluc1g/l
TMB3712 gluc20g/l
TMB3713 gluc1g/l
TMB3713 gluc20g/l
Fold change (relative to 0h)
Shake flask
Microplate
0h
3h
6h

B.

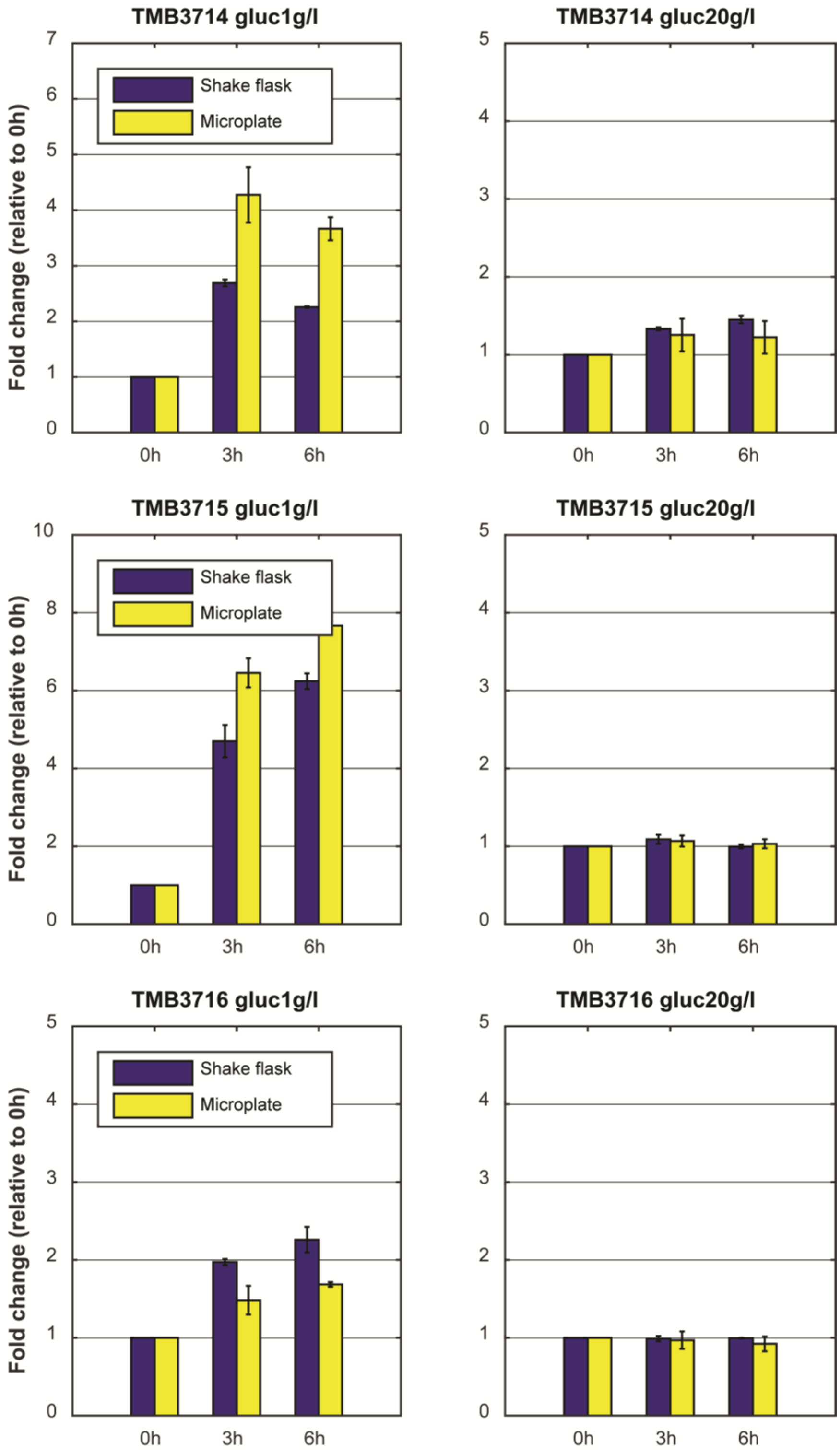
TMB3714 gluc1g/l
TMB3714 gluc20g/l
TMB3715 gluc1g/l
TMB3715 gluc20g/l
TMB3716 gluc1g/l
TMB3716 gluc20g/l
Fold change (relative to 0h)
Shake flask
Microplate
0h
3h
6h

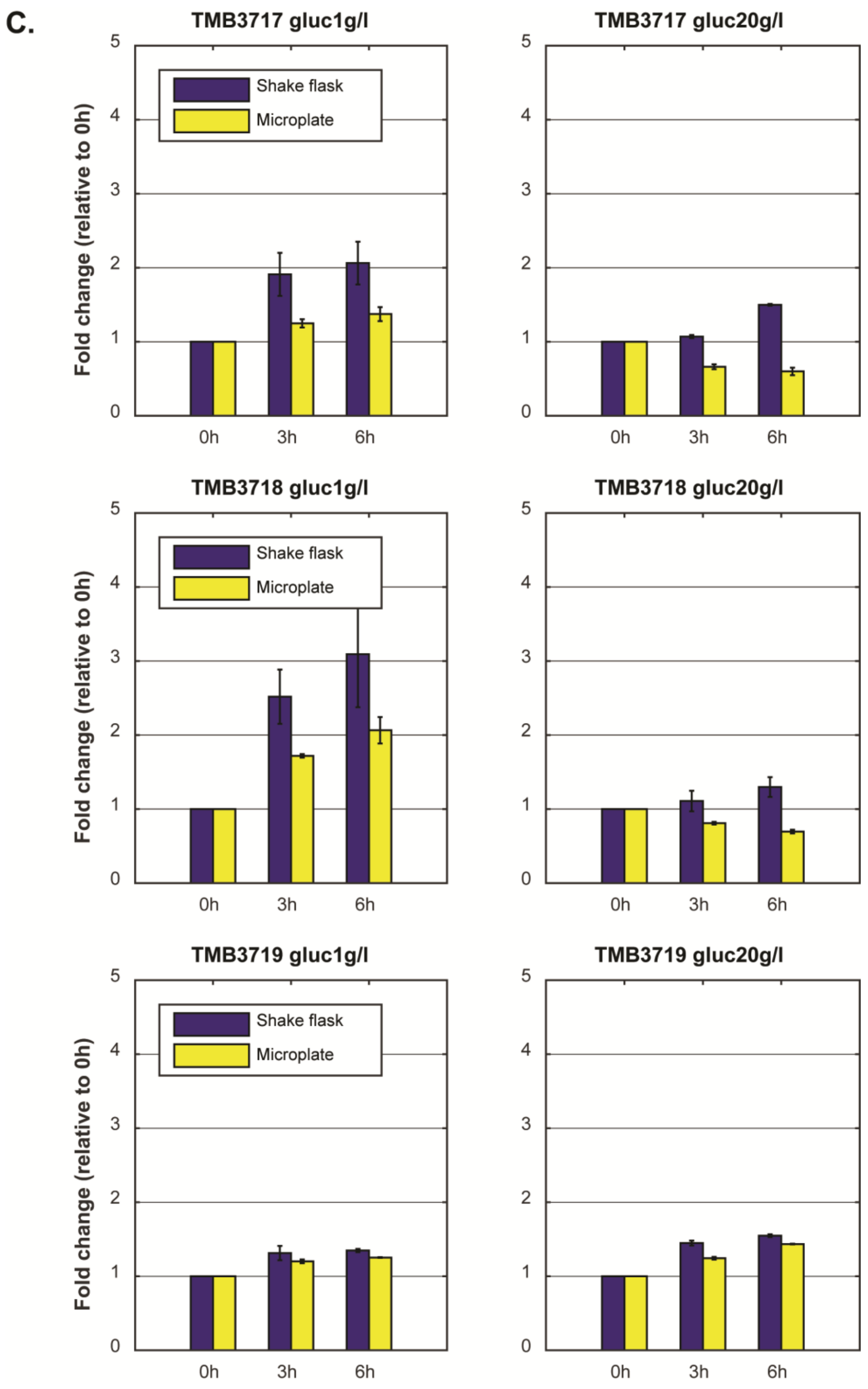

Figure S7. Comparison the FI results from the shake flask cultivations and the corresponding microplate cultivations, in terms of fold change relative to 0h. Data from Figure 2 & 3 in the main paper was converted to fold change (blue bars) for the glucose 1 g/l and 20 g/l conditions for each biosensor strain (A-C). This is presented alongside the data from Table D (yellow bars) for the 0h, 3h and 6h time points (as no other times were measured in the microtiter plate experiment).

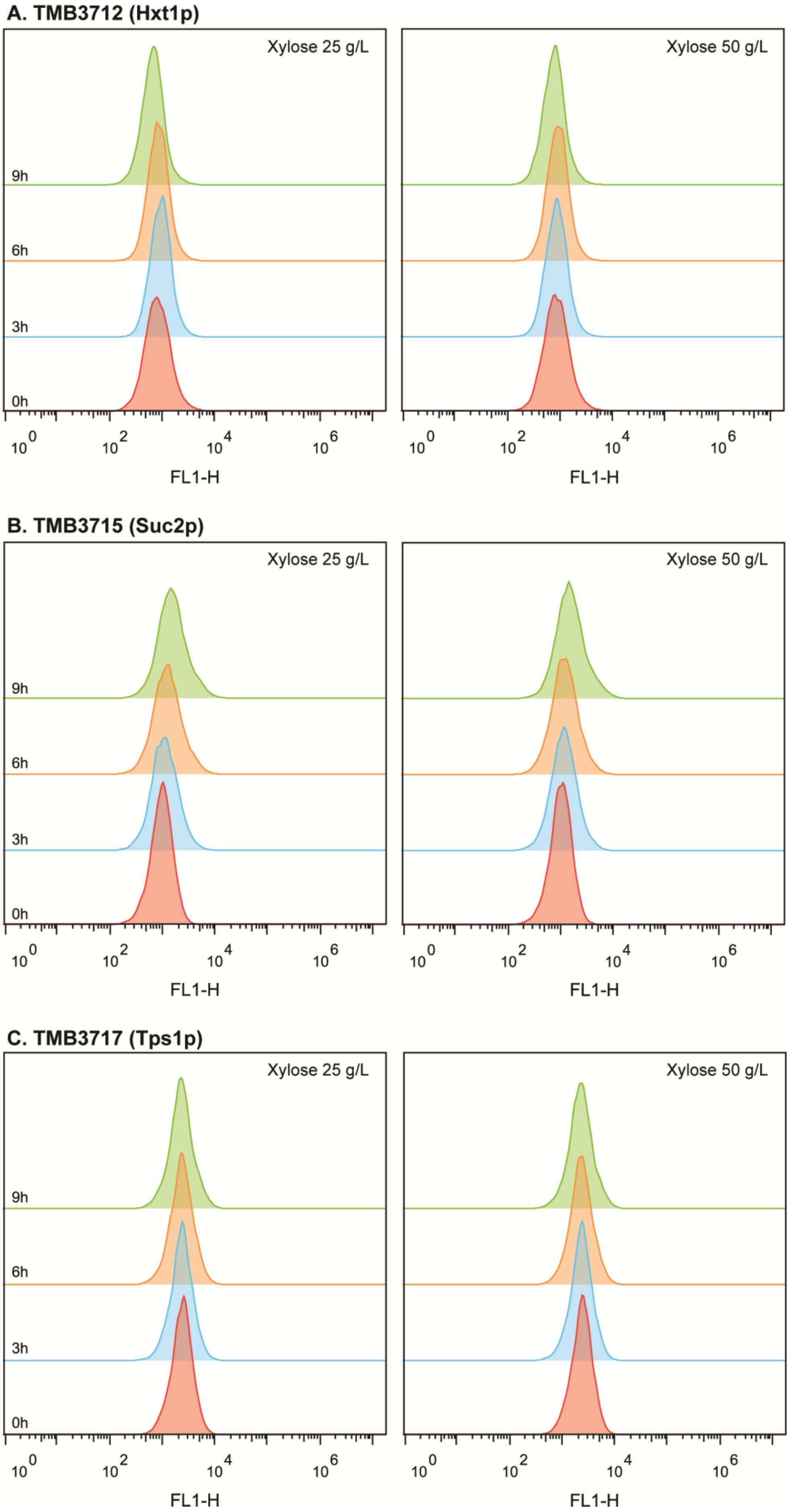

A. TMB3712 (Hxt1p)
Xylose 25 g/L
Xylose 50 g/L
9h
6h
3h
0h
10^0
10^2
10^4
10^6
FL1-H
B. TMB3715 (Suc2p)
C. TMB3717 (Tps1p)

Figure S8. Selected histograms from the data of Supplementary Table D. The subfigures show the FI signal distribution over 9h during two different xylose conditions (25 and 50 g/L). The histograms are representative of the distributions of strains TMB3712 and TMB3715-3719 and contrast the histograms of strains TMB3713-14 (Figure 6 in the main article) by not being affected by the presence of xylose.

Supplementary Tables

Table S1. Primers used in the present study. Underscored regions indicate introduced restriction enzyme sites. Lower case letters indicate tailing regions used for making targeting fragments.

Name	Description	Sequence	Reference
**TRP1_f**	Cloning of *TRP1*, forward primer	CTGTTATTAATTTCAC	[6]
**TRP1_r**	Cloning of *TRP1*, reverse primer	TCTTAGCATTTTTG	[6]
**HIS3_ flank_f**	Cloning of *HIS3*, forward primer	TATCGTTTGAACACGG	[6]
**HIS3_ flank_r**	Cloning of *HIS3*, reverse primer	AGTTCAGCCATAATATG	[6]
**ADE2_F2**	Verification of M3499 chromosomal integration	ATGGATTCTAGAACAGTTGGTATATTAG	[7]
**URA3_ CHR5_F627**	"	GGGAAGACAAGCAACGAAAC	This study
**yEGFP_ F1_KpnI**	Cloning of yEGFP3-PGK1t cassette with *KpnI* restriction site, forward primer	TGCGGTACCAAAAATGTCTAAAGCTGAAG	This study
**yEGFP_ R1_SacI**	Cloning of yEGFP3-PGK1t cassette with *SacI* restriction site, forward primer	TGAGCTCTGAACATAGAAATATCGAATG	This study
**HXT1p_f**	Cloning of *HXT1* promotor, forward primer with *SalI* site	TACGTCGACTAGCAGGGCGAGATTGGTGC	This study
**HXT1p_r**	Cloning of *HXT1* promotor, reverse primer with *BamHI* site	ATGGGATCCTGATTTTACGTATATCAACTA GTTGACGATTATG	This study
**HXT2p_f**	Cloning of *HXT2* promotor, forward primer with *SalI* site	ATCGTCGACCTATTTTACTTAAACGAAGAT AGGGTTTCGTAATC	This study
**HXT2p_r**	Cloning of *HXT2* promotor, reverse primer with *BamHI* site	ATGGGATCCTATGTTGCTTTATAA GTCTTTTTGTAAT	This study
**HXT4p_f**	Cloning of *HXT4* promotor, forward primer with *SalI* site	AACGTCGACCAACGATGTTGCC AAATAGTCGTACCTG	This study
**HXT4p_r**	Cloning of *HXT4* promotor, reverse primer with *BamHI* site	ATGGGATCCGGCAGATTTTATTGT AAAAGTGTTTCAAAACCAAAC	This study
**SUC2p_f**	Cloning of *SUC2* promotor, forward primer with *SalI* site	ATCGTCGACTTCCCAATG AACAAAGGACAGG	This study
**SUC2p_r**	Cloning of *SUC2* promotor, reverse primer with *BamHI* site	TTAGGATCCCATATACGTTAGTGA AAAGAAAAGCTTTTTGTTTTGC	This study
**CAT8p_f**	Cloning of *CAT8* promotor, forward primer with *SalI* site	AACGTCGACTAATATACGGCTCTA GCGTCACC	This study
**CAT8p_r**	Cloning of *CAT8* promotor, reverse primer	TGGGGATCCTTGTGTCTTCTCTTTTACTCA ACTTGTAAATTCTC	This study

	with *BamHI* site		
**TEF4p_f**	Cloning of *TEF4* promotor, forward primer with *SalI* site	TGCGTCGACCTTAATATCCTCTTCCTC TTCTTCATC	This study
**TEF4p_r**	Cloning of *TEF4* promotor, reverse primer with *BamHI* site	ATCGGATCCCTTGAATCTATCGAGGGC CAAAATC	This study
**TPS1p_f**	Cloning of *TPS1* promotor, forward primer with *SalI* site	TGAGTCGACGAATTTTACGATAGAGCC AGAGAC	This study
**TPS1p_r**	Cloning of *TPS1* promotor, reverse primer with *BamHI* site	ATCGGATCCAGTTCTATGTCTTAATAA GTCTGTATGTG	This study
**TPS2p_f**	Cloning of *TPS2* promotor, forward primer with *SalI* site	TGCGTCGACTAAACCAAGGAGTGCCCT CAGCGAAACCACTG	This study
**TPS2p_r**	Cloning of *TPS2* promotor, reverse primer with *BamHI* site	ACGGGATCCTTCGGCACAGAAATAGTG ACAGGCAGTGTTATTTTGG	This study
**CAN1_ Targ1_F**	Cloning of targeting fragment for introduction of the GFP plasmids in *CAN1*	GCTCTTTCCCGACGAGAGTAAATG	This study
**CAN1_ Targ1_Rtail**	"	ataaacaaataggggttccgcgcacatttccccgaaaagtgccac ctgacAAGAGGATGTAACAGGGATGAATG	This study
**CAN1_ Targ2_Ftail**	"	atacgcctatttttataggttaatgtcatgataataatggtttcttagac AACAAGTTGGCTCCTAAATTCCTG	This study
**CAN1_ Targ2_R**	"	CATAAATGTGGCCGCATAATAAGC	This study
**CAN1_ CHR5_F201**	Verification of chromosomal integration of reporter plasmids	CCGAATCAGGGAATCCCTTT	[8]
**AmpR_R130**	"	AATGATACCGCGAGACCCAC	This study
**URA3_R550**	"	TTGTACTTGGCGGATAATGCCTTTAG	This study
**CAN1_ CHR5_R129**	"	GCAAGATTGTTGTGGTGAATCATCG	This study; modified from [8]
**YIpGFP-prom_R2**	"	TTAAGGTCAATTTACCGTAAGTAGCATC	This study
**SPB1_ Targ1_F**	Cloning of targeting fragment for introduction of the YIplac128 plasmid in the *SPB1/PBN1* intergenic region	TTTGCCAGATTGGTTTTTAGAAG	This study
**SPB1_ Targ1_Rtail**	"	atttagaaaaataaacaaataggggttccgcgcacatttccccgaaaagt gccacctgacAAGGGAATGGAAAAATAATGCTC	This study; modified from [9]
**PBN1_ Targ2_Ftail**	"	tggcctcgtgatacgcctatttttataggttaatgtcatgataataatggtttct tagacATCATCAAAAAACTTATAGGAAACC	This study; modified from [9]
**PBN1_ Targ2_R**	"	CGAGATAAGGCATGGGGTTC	[9]
**SPB1_ verif_F**	Verification of chromosomal integration of the YIplac128 plasmid	AGGAAGAATGGACCGGTTTT	[9]
**PBN1_ verif_R**	"	GGAGGATGGACGATGGTAAA	[9]
**LEU2_R**	"	TTAAGCAAGGATTTTCTTAACTTCTTCG	This study

**ACT1_F**	RT-qPCR reference gene	TGGATTCCGGTGATGGTGTT	[10]
**ACT1_R**	"	TCAAAATGGCGTGAGGTAGAGA	[10]
**yEGFP3_ F1790_RT**	RT-qPCR assay of the yEGFP3 gene	TGGTGATGGTCCAGTCTTGTT	This study
**yEGFP3_ R1918_RT**	"	TGGGTAATACCAGCAGCAGT	This study
**SUC2_ F263_RT**	RT-qPCR assay of the *SUC2* gene	AACCCATTGCTATCGCTCCC	This study
**SUC2_ R397_RT**	"	AAGTCCAAATCGCAACGCAT	This study

Table S2. Characterization of some known regulatory elements of the *S. cerevisiae* promoters that were chosen for the sugar signaling study. The table was partially adapted from Weinhandl *et al.* [11]. Abbreviations - STRE: Stress response element; UAS: Upstream activating sequence; RGE: Rapid Growth Element.

Promoter	Binding protein and promoter regulatory motifs		References
HXT1p	Rgt1p:	-957 to -951; -921 to -915;	[12, 13]
		-772 to -766; -736 to -730;	
		-481 to -475; -451 to -445;	
		-399 to -393; -227 to -221	
	Mig1p:	-60 to 49	
HXT2p	Rgt1p:	-577 to -571; -430 to -424	[12]
		-393 to -387	
	Mig1p:	-427 to -415; -504 to -493	
	UAS:	-291 to-218	
HXT4p	Rgt1p:	-645 to -639; -424 to -418	[12, 14]
		-295 to -289	
	Mig1p:	-566 to -554; -498 to -486	
SUC2p	Mig1p/2p:	-499 to -480;-442 to -425	[15]
	Sko1p:	-627 to -617	
	UAS:	-650 to -418	
	TATA-box:	-133	
CAT8p	Mig1p:	-204 to -223	[16]
TPS1p	STRE:	-467; -354; -300;	[17]
		-273; -244; -233	
TPS2p	STRE:	-517; -484; -435;	[17]
		-415; -303	
TEF4p	Rgt1p:	-737 to -730	[12, 18]
	RGE:	-403 to -398	
	Tbf1p:	-187	

Table S3. Average maximum specific growth rate (μmax) of the biosensor strains during cultivation on glucose 20 g/L, including standard deviations. The cultivations were performed in biological duplicates. The corresponding growth curves can be found in Figures 3 and 4 in the main publication.

**Strain**	μ_{max} (h^{-1})
**TMB3711 (No GFP)**	0.34 ± 0.03
**TMB3712 (*HXT1p*)**	0.37 ± 0.01
**TMB3713 (*HXT2p*)**	0.36 ± 0.02
**TMB3714 (*HXT4p*)**	0.33 ± 0.01
**TMB3715 (*SUC2p*)**	0.35 ± 0.02
**TMB3716 (*CAT8p*)**	0.35 ± 0.03
**TMB3717 (*TPS1p*)**	0.32 ± 0.03
**TMB3718 (*TPS2p*)**	0.35 ± 0.01
**TMB3719 (*TEF4p*)**	0.37 ± 0.07

Table S4. Results of the microtiter-plate screening of the biosensors strains in terms of FI fold induction. The FI signal was normalized to the corresponding 0h signal of the given condition and strain. A value of 1 corresponds to repression (i.e. no fold change since time 0h). Values in bold indicate a fold change of >2.

Strain	YNB only (no carbon source)		Glucose 1 g/L		Glucose 20 g/L		Glucose 5 g/L	
	**3h**	**6h**	**3h**	**6h**	**3h**	**6h**	**3h**	**6h**
TMB3711 (No GFP)	0.92 ± 0.023	0.91 ±0.024	0.98 ±0.062	0.89 ±0.040	0.96 ±0.026	0.87 ±0.010	1.00 ±0.010	0.92 ±0.056
TMB3712 (*HXT1p*)	0.84 ±0.010	0.83 ±0.031	1.15 ±0.010	1.21 ±0.012	1.82 ±0.01	**4.14 ±0.17**	1.21 ±0.016	1.23 ±0.013
TMB3713 (*HXT2p*)	1.13 ±0.034	1.28 ±0.091	**3.62 ±0.130**	**3.01 ±0.068**	0.75 ±.013	0.67 ±0.021	1.10 ±0.264	1.34 ±0.11
TMB3714 (*HXT4p*)	1.04 ±0.088	1.15 ±0.18	**4.27 ±0.50**	**3.67 ±0.21**	1.25 ±0.21	1.22 ±0.21	1.77 ±0.058	**2.47 ±0.22**
TMB3715 (*SUC2p*)	1.10 ±0.22	1.44 ±0.56	**6.45 ±0.37**	**7.66 ±0.089**	1.07 ±0.071	1.03 ±0.059	0.98 ±0.15	**3.51 ±0.75**
TMB3716 (*CAT8p*)	1.01 ±0.010	1.27 ±0.041	1.48 ±0.18	1.69 ±0.030	0.97 ±0.11	0.92 ±0.094	0.98 ±0.002	1.14 ±0.039
TMB3717 (*TPS1p*)	1.04 ±0.001	1.08 ±0.018	1.25 ±0.056	1.37 ± 0.095	0.66 ±0.033	0.60 ±0.051	0.74 ±0.051	1.23 ±0.075
TMB3718 (*TPS2p*)	1.00 ±0.017	1.11 ±0.022	1.72 ±0.023	**2.06 ±0.18**	0.81 ±0.018	0.70 ±0.024	0.85 ±0.026	1.61 ±0.084
TMB3719 (*TEF4p*)	0.95 ±0.025	1.01 ±0.043	1.20 ±0.025	1.25 ±0.010	1.24 ±0.018	1.44 ±0.010	1.35 ±0.013	1.46 ±0.010

Strain	Glycerol 3% (v/v)		Xylose 50 g/L		Xylose 50 g/L + Gluc. 5 g/L		Xyl. 50 g/L + Glyc. 3% (v/v)	
	**3h**	**6h**	**3h**	**6h**	**3h**	**6h**	**3h**	**6h**
TMB3711 (No GFP)	0.82 ±0.024	0.79 ±0.017	0.79 ±0.017	0.78 ±0.023	1.04 ±0.010	1.02 ±0.041	0.76 ±0.014	0.72 ±0.013
TMB3712 (*HXT1p*)	0.75 ±0.013	0.79 ±0.051	0.83 ±0.015	0.84 ±0.010	1.48 ±0.011	1.70 ±0.011	1.22 ±0.097	1.14 ±0.064
TMB3713 (*HXT2p*)	1.09 ±0.037	1.28 ±0.064	0.89 ±0.039	0.89 ±0.046	1.37 ±0.26	**2.01 ±0.55**	0.89 ±0.012	0.91 ±0.009
TMB3714 (*HXT4p*)	0.98 ±0.087	1.06 ±0.16	1.08 ±0.25	1.31 ±0.51	**2.15 ±0.31**	**3.45 ±0.62**	0.96 ±0.14	1.09 ±0.33
TMB3715 (*SUC2p*)	0.98 ±0.20	1.20 ±0.40	0.95 ±0.12	1.06 ±0.21	1.13 ±0.10	**5.15 ±0.62**	0.91 ±0.17	1.02 ±0.27
TMB3716 (*CAT8p*)	0.89 ±0.027	1.09 ±0.049	0.82 ±0.003	0.84 ±0.018	1.08 ±0.037	1.13 ±0.037	0.80 ±0.004	0.78 ±0.009
TMB3717 (*TPS1p*)	0.92 ±0.006	0.91 ±0.029	0.92 ± 0.006	0.91 ±0.029	0.70 ±0.048	0.77 ±0.047	0.91 ±0.007	0.91 ±0.026
TMB3718 (*TPS2p*)	0.93 ±0.020	1.03 ±0.029	0.85 ±0.012	0.85 ±0.026	0.95 ±0.076	1.05 ±0.032	0.84 ±0.012	0.82 ±0.025
TMB3719 (*TEF4p*)	0.97 ±0.015	1.07 ±0.040	0.87 ±0.012	0.96 ±0.010	1.28 ±0.018	1.42± 0.0032	0.87 ±0.010	0.95 ±0.0027

Table S5. Effect of different concentrations of xylose on the biosensor strains. The effect of xylose was investigated in terms of FI fold induction (normalized to 0h for the given condition and strain), as well as prolonged cultivation time (9h; cf. Table 3 in the main article). A value of 1 corresponds to repression (i.e. no fold change since time 0h).

Strain	YNB only (no carbon source)			Xylose 25 g/L			Xylose 50 g/L		
	**3h**	**6h**	**9h**	**3h**	**6h**	**9h**	**3h**	**6h**	**9h**
**TMB3711 (No GFP)**	0.92 ±0.01	1.00 ±0.03	1.07 ±0.10	0.91 ±0.04	0.96 ±0.01	1.00 ±0.01	0.90 ±0.01	0.92 ±0.02	0.94 ±0.03
**TMB3712 (*HXT1p*)**	1.03 ±0.02	0.84 ±0.01	0.79 ±0.01	1.32 ±0.26	1.20 ±0.22	1.19 ±0.50	1.21 ±0.26	1.12 ±0.20	1.00 ±0.24
**TMB3713 (*HXT2p*)**	1.00 ±0.02	1.03 ±0.07	0.97 ±0.05	1.20 ±0.046	1.76 ±0.01	2.35 ±0.06	1.21 ±0.02	1.72 ±0.04	2.25 ±0.03
**TMB3714 (*HXT4p*)**	0.99 ±0.01	1.04 ±0.03	1.02 ±0.03	1.36 ±0.10	2.34 ±0.05	2.94 ±0.22	1.33 ±0.11	1.88 ±0.17	2.78 ±0.02
**TMB3715 (*SUC2p*)**	1.04 ±0.06	1.12 ±0.01	1.21 ±0.09	1.06 ±0.11	1.18 ±0.14	1.22 ±0.09	1.06 ±0.12	1.12 ±0.12	1.17 ±0.10
**TMB3716 (*CAT8p*)**	1.12 ±0.14	1.22 ±0.10	1.31 ±0.03	1.07 ±0.10	1.19 ±0.17	1.26 ±0.19	1.04 ±0.070	1.09 ±0.09	1.16 ±0.13
**TMB3717 (*TPS1p*)**	0.97 ±0.08	0.92 ±0.001	0.90 ±0.02	0.97 ±0.05	0.99 ±0.08	0.95 ±0.06	0.97 ±0.04	0.97 ±0.042	0.94 ±0.05
**TMB3718 (*TPS2p*)**	1.07 ±0.08	1.08 ±0.002	1.08 ±0.07	1.04 ±0.05	1.06 ±0.007	1.08 ±0.012	1.03 ±0.05	1.00 ±0.02	1.02 ±0.02
**TMB3719 (*TEF4p*)**	0.93 ±0.004	0.74 ±0.01	0.60 ±0.01	1.04 ±0.04	0.96 ±0.008	0.85 ±0.032	0.95 ±0.02	1.00 ±0.02	0.88 ±0.02

Strain	Xylose 75 g/L			Xylose 100 g/L		
	**3h**	**6h**	**9h**	**3h**	**6h**	**9h**
**TMB3711 (No GFP)**	0.89 ±0.01	0.90 ±0.04	0.91 ±0.06	0.88 ±0.01	0.88 ±0.05	0.89 ±0.07
**TMB3712 (*HXT1p*)**	1.22 ±0.12	1.02 ±0.06	0.97 ±0.07	1.25 ±0.07	1.12 ±0.01	1.09 ±0.02
**TMB3713 (*HXT2p*)**	1.17 ±0.02	1.63 ±0.01	1.91 ±0.027	1.09 ±0.04	1.31 ±0.01	1.51 ±0.03
**TMB3714 (*HXT4p*)**	1.27 ±0.07	1.56 ±0.03	1.97 ±0.11	1.15 ±0.04	1.25 ±0.02	1.36 ±0.01
**TMB3715 (*SUC2p*)**	1.03 ±0.09	1.06 ±0.06	1.10 ±0.03	1.01 ±0.07	1.03 ±0.05	1.04 ±0.02
**TMB3716 (*CAT8p*)**	1.01 ±0.03	1.04 ±0.03	1.04 ±0.03	0.98 ±0.01	1.00 ±0.01	0.99 ±0.013
**TMB3717 (*TPS1p*)**	0.98 ±0.04	0.94 ±0.02	0.88 ±0.006	0.98 ±0.04	0.94 ±0.01	0.84 ±0.007
**TMB3718 (*TPS2p*)**	1.01 ±0.03	0.97 ±0.06	0.93 ±0.05	1.00 ±0.03	0.96 ±0.05	0.92 ±0.07
**TMB3719 (*TEF4p*)**	0.90 ±0.03	0.93 ±0.07	0.92 ±0.02	0.86 ±0.012	0.86± 0.03	0.90 ±0.07

References

1. Knijnenburg TA, Roda O, Wan YK, Nolan GP, Aitchison JD, Shmulevich I. A regression model approach to enable cell morphology correction in high-throughput flow cytometry. Mol Syst Biol. 2011;7(531). doi: 10.1038/msb.2011.64. PubMed PMID: 21952134.

2. Bauminger BB. Micro Method for Manual Analysis of True Glucose in Plasma without Deproteinization. J Clin Pathol. 1974;27(12):1015-7. doi: 10.1136/jcp.27.12.1015. PubMed PMID: 4452743.

3. Harkness TAA, Arnason TG. A Simplified Method for Measuring Secreted Invertase Activity in *Saccharomyces cerevisiae*. Biochemistry & Pharmacology: Open Access. 2014;3(6):151. doi: 10.4172/2167-0501.1000151.

4. Weiß P, Huppert S, Kolling R. ESCRT-III Protein Snf7 Mediates High-Level Expression of the SUC2 Gene via the Rim101 Pathway. Eukaryot Cell. 2008;7(11):1888-94. doi: 10.1128/Ec.00194-08. PubMed PMID: 18806212.

5. Kayyali US, Moore TB, Randall JC, Richardson RJ. Neurotoxic Esterase (Nte) Assay - Optimized Conditions Based on Detergent-Induced Shifts in the Phenol/4-Aminoantipyrine Chromophore Spectrum. J Anal Toxicol. 1991;15(2):86-9. PubMed PMID: 2051750.

6. Knudsen JD, Carlquist M, Gorwa-Grauslund M. NADH-dependent biosensor in *Saccharomyces cerevisiae*: principle and validation at the single cell level. AMB Express. 2014;4(1):81. doi: 10.1186/s13568-014-0081-4. PubMed PMID: 25401080.

7. Tokuhiro K, Muramatsu M, Ohto C, Kawaguchi T, Obata S, Muramoto N, Hirai M, Takahashi H, Kondo A, Sakuradani E, Shimizu S. Overproduction of Geranylgeraniol by Metabolically Engineered *Saccharomyces cerevisiae*. Appl Environ Microb. 2009;75(17):5536-43. doi: 10.1128/Aem.00277-09. PubMed PMID: 19592534.

8. Solis-Escalante D, van den Broek M, Kuijpers N, Pronk J, Boles E, Daran J, Daran-Lapujade P. The genome sequence of the popular hexose-transport-deficient *Saccharomyces cerevisiae*strain EBY. VW4000 reveals LoxP/Cre-induced translocations and gene loss. FEMS Yeast Res. 2015;15(2). doi: 10.1093/femsyr/fou004. PubMed PMID: 25673752.

9. Flagfeldt DB, Siewers V, Huang L, Nielsen J. Characterization of chromosomal integration sites for heterologous gene expression in *Saccharomyces cerevisiae*. Yeast. 2009;26(10):545-51. doi: 10.1002/Yea.1705. PubMed PMID: 19681174.

10. Ismail KSK, Sakamoto T, Hatanaka H, Hasunuma T, Kondo A. Gene expression cross-profiling in genetically modified industrial *Saccharomyces cerevisiae* strains during high-temperature ethanol production from xylose. J Biotechnol. 2013;163(1):50-60. doi: 10.1016/j.jbiotec.2012.10.017. PubMed PMID: 23131464.

11. Weinhandl K, Winkler M, Glieder A, Camattari A. Carbon source dependent promoters in yeasts. Microb Cell Fact. 2014;13(5). doi: 5 10.1186/1475-2859-13-5. PubMed PMID: 24401081.

12. Kim JH. DNA-binding properties of the yeast Rgt1 repressor. Biochimie. 2009;91(2):300-3. doi: 10.1016/j.biochi.2008.09.002. PubMed PMID: 18950675.

13. Sinha S, Tompa M. Discovery of novel transcription factor binding sites by statistical overrepresentation. Nucleic Acids Res. 2002;30(24):5549-60. doi: Doi 10.1093/Nar/Gkf669. PubMed PMID: 12490723.

14. Özcan S, Johnston M. Two different repressors collaborate to restrict expression of the yeast glucose transporter genes HXT2 and HXT4 to low levels of glucose. Mol Cell Biol. 1996;16(10):5536-45. doi: 10.1128/MCB.16.10.5536. PubMed PMID: 8816466.

15. Bu Y, Schmidt MC. Identification of cis-acting elements in the SUC2 promoter of Saccharomyces cerevisiae required for activation of transcription. Nucleic Acids Res. 1998;26(4):1002-9. doi: DOI 10.1093/nar/26.4.1002. PubMed PMID: 9461460.

16. Randez-Gil F, Bojunga N, Proft M, Entian KD. Glucose derepression of gluconeogenic enzymes in Saccharomyces cerevisiae correlates with phosphorylation of the gene activator Cat8p. Mol Cell Biol. 1997;17(5):2502-10. doi: 10.1128/MCB.17.5.2502 PubMed PMID: 9111319.

17. Moskvina E, Schuller C, Maurer CTC, Mager WH, Ruis H. A search in the genome of Saccharomyces cerevisiae for genes regulated via stress response elements. Yeast. 1998;14(11):1041-50. doi: 10.1002/(Sici)1097-0061(199808)14:11<1041::Aid-Yea296>3.0.Co;2-4. PubMed PMID: 9730283.

18. Bosio MC, Negri R, Dieci G. Promoter architectures in the yeast ribosomal expression program. Transcription. 2011;2(2):71-7. doi: 10.4161/trns.2.2.14486. PubMed PMID: 21468232.
